# Causal effects of specific gut microbiota on musculoskeletal diseases: a bidirectional two-sample Mendelian randomization study

**DOI:** 10.3389/fmicb.2023.1238800

**Published:** 2023-08-17

**Authors:** Shuai Chen, Huawei Han, Xiaohe Sun, Guowei Zhou, Qing Zhou, Zhiwei Li

**Affiliations:** ^1^Department of Orthopaedics, The Second Affiliated Hospital of Nanjing University of Chinese Medicine, Nanjing, China; ^2^Department of Oncology, Jiangsu Province Hospital of Chinese Medicine, Affiliated Hospital of Nanjing University of Chinese Medicine, Nanjing, China; ^3^Department of General Surgery, Jiangsu Province Hospital of Chinese Medicine, Affiliated Hospital of Nanjing University of Chinese Medicine, Nanjing, China; ^4^Department of Ophthalmology, Children’s Hospital of Nanjing Medical University, Nanjing, China

**Keywords:** musculoskeletal diseases, gut microbiota, Mendelian randomization, sarcopenia, causality

## Abstract

**Background:**

Recent observational studies and clinical trials demonstrated an association between gut microbiota and musculoskeletal (MSK) diseases. Nonetheless, whether the gut microbiota composition has a causal effect on the risk of MSK diseases remains unclear.

**Methods:**

Based on large-scale genome-wide association studies (GWAS), we performed a two-sample Mendelian randomization (MR) analysis to investigate the causal relationship between gut microbiota and six MSK diseases, namely osteoporosis (OP), fracture, sarcopenia, low back pain (LBP), rheumatoid arthritis (RA), and ankylosing spondylitis (AS). Instrumental variables for 211 gut microbiota taxa were obtained from the largest available GWAS meta-analysis (*n* = 18,340) conducted by the MiBioGen consortium. And the summary-level data for six MSK diseases were derived from published GWAS. The inverse-variance weighted (IVW) method was conducted as a primary analysis to estimate the causal effect, and the robustness of the results was tested via sensitivity analyses using multiple methods. The Bonferroni-corrected test was used to determine the strength of the causal relationship between gut microbiota and various MSK diseases. Finally, a reverse MR analysis was applied to evaluate reverse causality.

**Results:**

According to the IVW method, we found 57 suggestive causal relationships and 3 significant causal relationships between gut microbiota and MSK diseases. Among them, *Genus Bifidobacterium* (β: 0.035, 95% CI: 0.013–0.058, *p* = 0.0002) was associated with increased left handgrip strength, *Genus Oxalobacter* (OR: 1.151, 95% CI: 1.065–1.245, *p* = 0.0003) was correlated with an increased risk of LBP, and *Family Oxalobacteraceae* (OR: 0.792, 95% CI: 0.698–0.899, *p* = 0.0003) was linked with a decreased risk of RA. Subsequently, sensitivity analyses revealed no heterogeneity, directional pleiotropy, or outliers for the causal effect of specific gut microbiota on MSK diseases (*p* > 0.05). Reverse MR analysis showed fracture may result in a higher abundance of *Family Bacteroidales* (*p* = 0.030) and sarcopenia may lead to a higher abundance of *Genus Sellimonas* (*p* = 0.032).

**Conclusion:**

Genetic evidence suggested a causal relationship between specific bacteria taxa and six MSK diseases, which highlights the association of the “gut-bone/muscle” axis. Further exploration of the potential microbiota-related mechanisms of bone and muscle metabolism might provide novel insights into the prevention and treatment of MSK diseases.

## 1. Introduction

Musculoskeletal (MSK) diseases encompass a spectrum of pathologies that affect muscles, soft tissues, joints and the spine (Murray et al., 2012). Globally, all MSK diseases combined accounted for 21.3% of the total years lived with disability (YLDs), second only to mental and behavioral disorders (23.2%) (March et al., 2014; Smith et al., 2014). Disability due to MSK diseases has increased by 45% from 1990 to 2010, in particular sarcopenia, osteoporosis (OP) and osteoarthritis (OA), and is expected to continue to rise with an increasingly obese, sedentary, and ageing population (Vos et al., 2012). In addition, MSK diseases also impose a significant economic burden on both patients and the health care system (Sebbag et al., 2019). Recent epidemiological studies have revealed that MSK diseases-related lost productivity accounts for 2% of the gross domestic product (GDP) in the European Union (Bevan, 2015). According to data from the 2012 National Health Interview Survey (NHIS), the annual cost of direct treatment and lost wages for MSK diseases in the United States was estimated at $213 billion (Rosenfeld et al., 2018). Therefore, the prevention and management of MSK diseases has been globally recognized as a crucial public health issue that needs to be solved urgently.

The gut microbiota is a large and complex community of microbial species inhabiting the human gastrointestinal tract, which plays a critical role in human health and diseases (Li et al., 2022). Human gut microbiota can influence host physiology by regulating multiple processes, including inflammation, oxidative stress, immune function, and anabolic balance (Ticinesi et al., 2019). Growing evidence suggests that many MSK diseases, such as OP, OA, sarcopenia, and low back pain (LBP) are closely correlated with altered gut microbiota (Biver et al., 2019). According to a recent study by Xu et al., the gut microbiota compositions of patients with osteoporosis were significantly different from those of healthy controls, especially the enriched *Dialister* and *Faecalibacterium* genera (Xu et al., 2020). Another observational study indicated that the abundance of *Lactobacilli*, *Bacteroides*, and *Prevotella* is higher and the abundance of *Enterobacteriaceae* is lower in normal individuals than in those with sarcopenia (Castro-Mejia et al., 2020). In addition, the gut microbiota can also be involved in the development of rheumatoid arthritis (RA) and ankylosing spondylitis (AS) by altering intestinal barrier permeability, regulating expression levels of host hormones, and mediating immune responses (Asquith et al., 2019). Although the correlations between gut microbiota and Musculoskeletal (MSK) diseases have been reported by previous researches, these findings are mainly based on observational and cross-sectional analyses, and it is still unclear whether there is a causal link between the gut microbiome and MSK diseases.

Mendelian randomization (MR) is a groundbreaking analytical method that provides an unbiased estimation of the causal link between phenotypes (Smith and Ebrahim, 2003). Compared with traditional observational studies, the MR study uses genetic variation as instrumental variables (IVs) to avoid the influence of traditional confounding factors and reverse causality, which provides robust evidence on the mechanisms of disease pathogenesis and the efficacy of treatments (Davey and Hemani, 2014). Given the aforementioned findings and the MR method, our objective was to conduct a bidirectional two-sample MR analysis using extensive genome-wide association study (GWAS) data to explore the causality between specific gut microbiota and six MSK diseases, including OP, fracture, sarcopenia, LBP, RA, and AS.

## 2. Materials and methods

### 2.1. Study design

**Figure 1:**
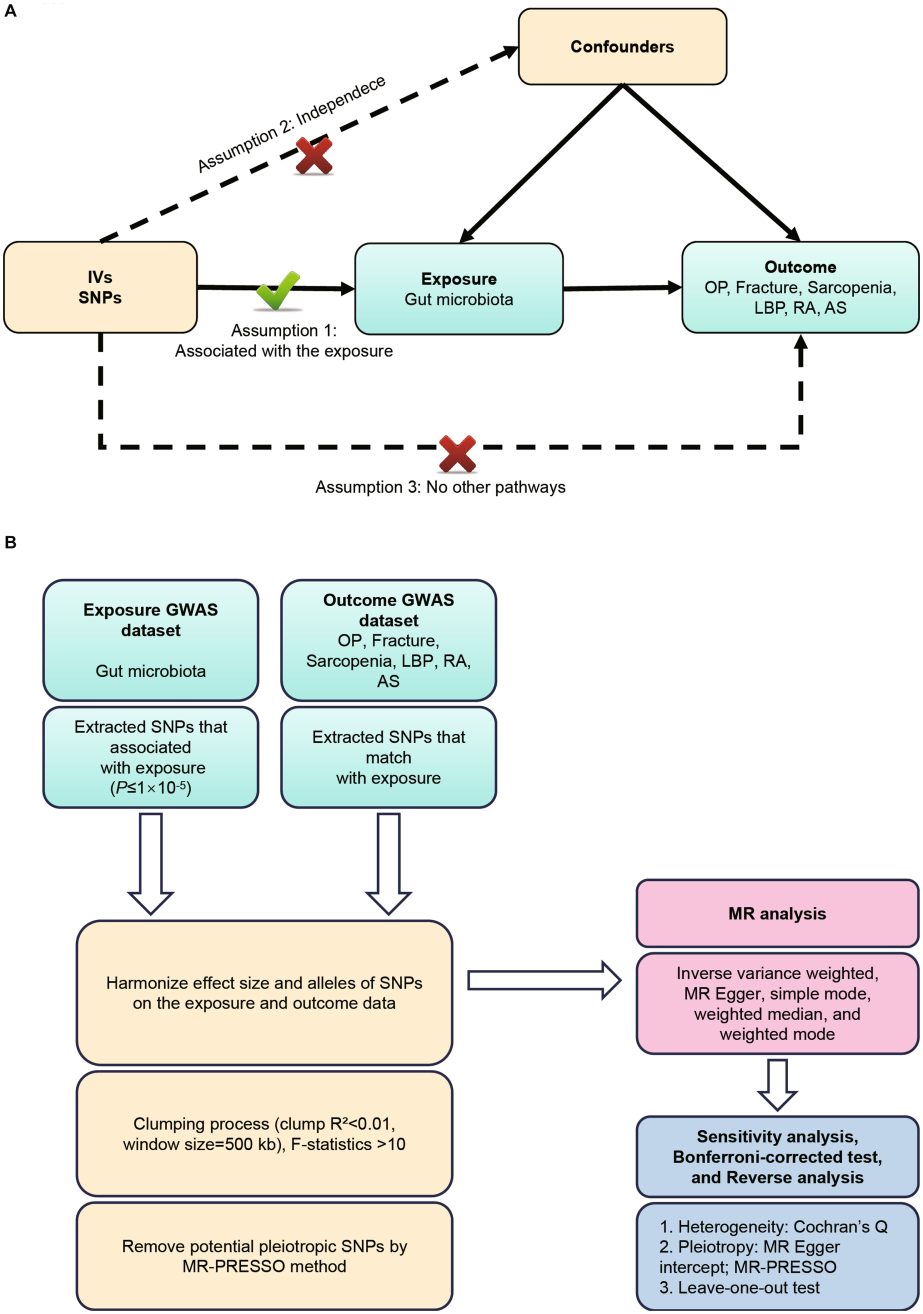
**(A)** Three assumptions of Mendelian randomization. **(B)** Flowchart of this Mendelian randomization study. MR, Mendelian randomization; MSK, musculoskeletal; OP, osteoporosis; LBP, low back pain; RA, rheumatoid arthritis; AS, ankylosing spondylitis.

GWAS summary data focusing on the relationships between genetics and disease have been used to detect a causal relationship between the gut microbiome and six MSK diseases (OP, fracture, sarcopenia, LBP, RA, and AS) through bidirectional two-sample MR analysis (Visscher et al., 2012; Jia et al., 2019). Our first step was to determine whether the gut microbiome contributes to the prevention or promotion of six MSK diseases, by selecting the gut microbiome as the exposure and the six MSK diseases as the outcome. Furthermore, we examined changes in gut microbiota following the development of six MSK diseases. According to Bownden et al., the two-sample MR needs to satisfy the following three assumptions (Bowden et al., 2015): (Murray et al., 2012) The instruments of genetic variations should be robustly associated with gut microbiota; (b) The genetic variations should not be associated with any confounders of gut microbiota and six MSK diseases; (c) The genetic variations should affect six MSK diseases solely through gut microbiota, not via other pathways (Sanderson et al., 2019) (Figure 1A). All studies included in the cited GWASs were approved by relevant review committees and all participants provided informed consent. The flow chart of the MR analysis was presented in Figure 1B.

### 2.2. Genome-wide association studies sources

Our gut microbiota summary data came from a large multi-ethnic GWAS meta-analysis that included 18,340 European individuals and individuals from 24 cohorts (MiBioGen Consortium). Three different variable regions of the 16S rRNA gene were targeted in order to profile the microbial composition. And only the taxa found in >10% of the samples were included in the quantitative microbiome trait loci (mbQTL) mapping study for each cohort. Furthermore, after adjustment for age, sex, technical covariates, and genetic principal components, Spearman’s correlation analysis was conducted to identify genetic loci that affected the covariate-adjusted abundance of bacterial taxa (Kurilshikov et al., 2021).

In the study, we extracted GWAS summary statistics for six MSK diseases from publicly GWAS analyses. The GWAS summary data on OP were mainly extracted from UK Biobank, including 7,547 cases and 455,386 controls of European ancestry (Sudlow et al., 2015). Summary statistics for fracture were derived from a GWAS meta-analysis, including 44,502 cases and 415,887 controls of European ancestry (Morris et al., 2019). Summary data for ALM and handgrip strength were obtained from the UK Biobank study. An analysis of 450,243 UK Biobank cohort participants was conducted to quantify ALM-related values by summing fat-free mass (Pei et al., 2020). For handgrip strength, UK Biobank provided GWAS summary statistics on right and left handgrip strength based on 461,089 and 461,026 United Kingdom people, respectively (Sudlow et al., 2015). The data on LBP came from a meta-analysis including 13,178 cases and 164,682 controls of European ancestry (https://gwas.mrcieu.ac.uk/datasets/ukb-d-M13_LOWBACKPAIN). For RA, we collected summary statistics from a GWAS meta-analysis that includes 14,361 RA cases and 43,923 controls of European ancestry (Okada et al., 2014). The genetic data of AS risk were derived from a large GWAS involving 9,069 cases and 1,550 controls of European (Cortes et al., 2013). Detailed information on the demographic characteristics of selected summary-level GWASs applied in this study was shown in Supplementary Table S1.

### 2.3. Selection of genetic instrumental variables

During the selection of the optimal IVs, the following quality control steps were taken to ensure the validity and accuracy of the conclusions on the causal relationship between the gut microbiome and the six MSK diseases (Murray et al., 2012) The instrumental variables selected for analysis need to be highly related to the corresponding exposures (We choose significant SNPs based on a loose cutoff of *p* < 1 × 10^−5^ to ensure sufficient instrumental variables for screening). (March et al., 2014) The instrumental variables are mutually independent and avoid the offset caused by linkage disequilibrium (LD) between the SNPs (r^2^ < 0.01, LD distance = 500 kb). (Smith et al., 2014) We eliminated instrumental variables with an F-statistic less than 10 to minimize potential weak instrument bias F = R^2^(n-k-1)/k(1-R^2^) (n is the sample size, k is the number of included instrumental variables, and R^2^ is the exposure variance explained by the selected SNPs).

### 2.4. Statistical analysis

The inverse variance weighted (IVW) method was employed as the main analysis, to obtain an unbiased estimate of the causal relationship between gut microbiota and MSK diseases. Furthermore, the weighted median, MR Egger, simple mode, and weighted mode methods were applied as additional methods to estimate causal effects under different conditions. The weighted median method could combine data on multiple genetic variants into a single causal estimate and provide a consistent estimate if at least half of the weight is derived from valid IVs (Bowden et al., 2016). The MR-Egger method could evaluate whether genetic variants have directional pleiotropy and provide a consistent estimate of the causal effect (Bowden et al., 2015). The intercept of MR-Egger regression was calculated to assess horizontal pleiotropy, and *p* value >0.05 indicated that the possibility of pleiotropy effect in the causal analysis is weak (Verbanck et al., 2018). Cochran’s Q test was derived from IVW estimation and used to detect heterogeneity among instrumental variables (Pierce and Burgess, 2013). In addition, we applied the Mendelian randomization pleiotropy residual sum and outlier (MR-PRESSO) method to determine horizontal pleiotropy and correct potential outliers. The leave-one-out method was used for sensitivity analysis, which sequentially removed one of the SNPs and used the remaining SNPs as instrumental variables for two-sample MR analysis to judge the degree of influence of causal association effect by a single SNP. In addition, we also performed a reverse-direction MR analysis to determine whether there was a reverse-direction causal relationship.

To obtain a more accurate assessment of the causal relationship, we applied Bonferroni correction to determine the significance of multiple-testing at each feature level (*p* < 0.05/n, where n is the number of bacterial taxa included in each feature level). Hence, the multiple-testing significance was 5.56 × 10^−3^, 3.13 × 10^−3^, 2.50 × 10^−3^, 1.56 × 10^−3^, and 4.20 × 10^−4^, respectively, for phylum, class, order, family, and genus. The ‘TwoSampleMR’ package and the ‘MRPRESSO’ package in R software (version 4.1.3) were used for all MR analyses.

## 3. Results

### 3.1. Selection of instrumental variables

To begin with, 14,587 SNPs correlated with the gut microbiota were identified as IVs from the MiBioGen Consortium at a relatively loose significance level (*p* < 1 × 10^−5^). It contained 211 bacterial traits, including 131 genera, 35 families, 20 orders, 16 classes, and 9 phyla. After a series of quality control steps, 2,236 SNPs were finally included in the analysis. In addition, the F-statistics of all IVs were >10, indicating no evidence of weak instrument bias.

### 3.2. Causal effects of gut microbiota on musculoskeletal diseases

#### 3.2.1. Causal effect of gut microbiota on osteoporosis risk

**Figure 2:**
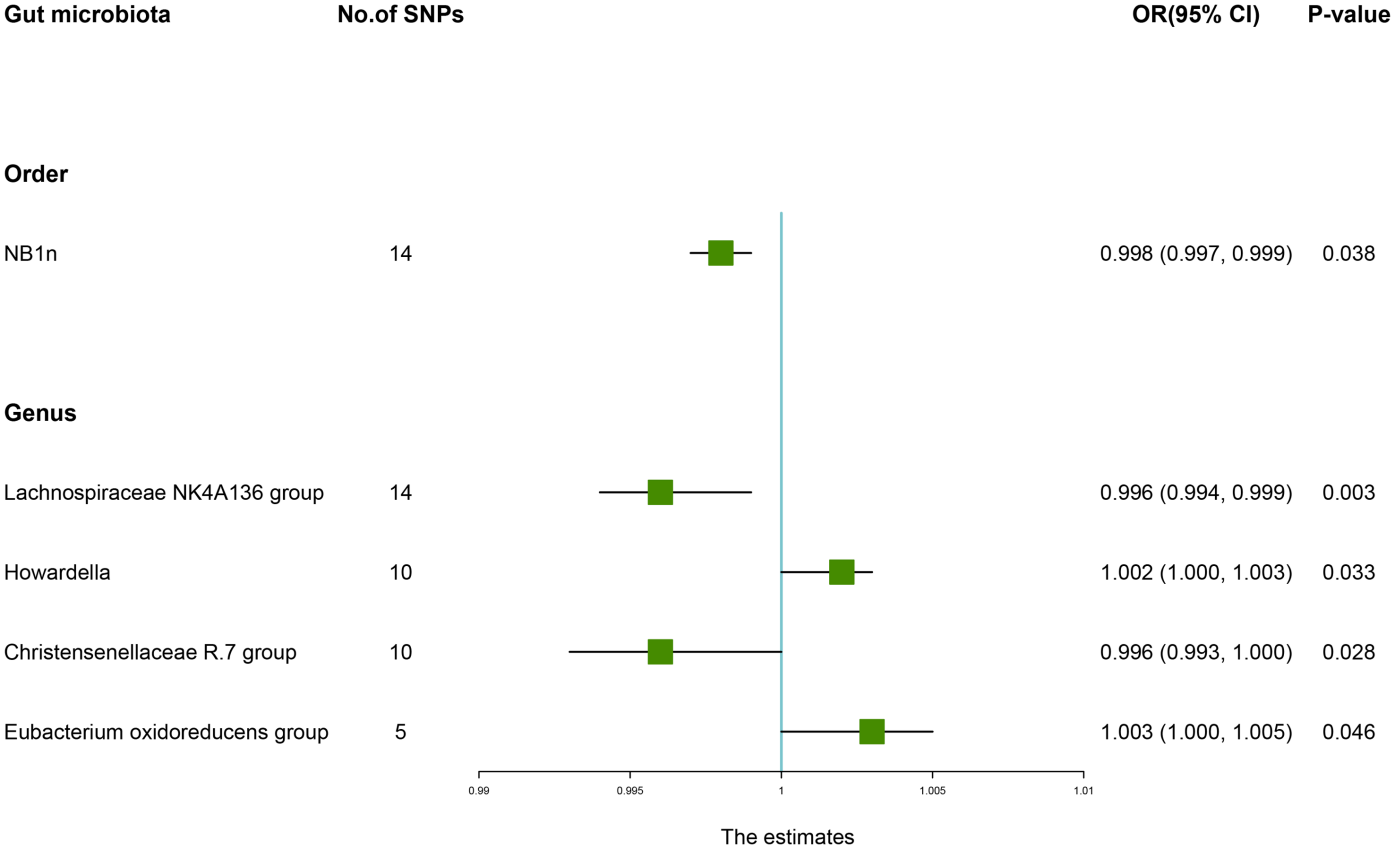
Forest plot of the causality between gut microbiota with the risk of osteoporosis. The estimates: Inverse variance weighted (IVW) results of gut microbiota and osteoporosis risk; *p-*value: *p-*value of the estimate. OR, odds ratio; SNP, single-nucleotide polymorphism.

According to the results of the IVW method, the higher genetically predicted abundance of *Order NB1n* (OR: 0.998, 95% CI: 0.997–0.999, *p* = 0.038), *Genus Lachnospiraceae NK4A136 group* (OR: 0.996, 95% CI: 0.994–0.999, *p* = 0.003), *Genus Christensenellaceae R.7 group* (OR: 0.996, 95% CI: 0.991–1.000, *p* = 0.028) was associated with a reduced risk of OP (Table 1). In contrast, genetically predicted abundance of *Genus Howardella* (OR: 1.002, 95% CI: 1.000–1.003, *p* = 0.033), and *Genus Eubacterium oxidoreducens group* (OR: 1.003, 95% CI: 1.000–1.005, *p* = 0.046) was positively related to OP risk (Figure 2).

**Table 1:** Mendelian randomisation (MR) results of causal effects between gut microbiome and the risk of osteoporosis.

Group	Bacterial traits	Nsnp	Methods	SE	OR (95% CI)	*p-*value
*Order*	*NB1n*	14	MR Egger	0.003	0.996 (0.990, 1.002)	0.214
			Weighted median	0.001	0.998 (0.997, 1.000)	0.103
			Inverse variance weighted	0.001	0.998 (0.997, 0.999)	0.038
			Simple mode	0.002	0.999 (0.996, 1.002)	0.412
			Weighted mode	0.002	0.999 (0.996, 1.002)	0.393
*Genus*	*Lachnospiraceae NK4A136 group*	14	MR Egger	0.003	0.991 (0.985, 0.997)	0.013
			Weighted median	0.002	0.997 (0.994, 1.001)	0.177
			Inverse variance weighted	0.001	0.996 (0.994, 0.999)	0.003
			Simple mode	0.003	0.998 (0.992, 1.004)	0.576
			Weighted mode	0.004	0.998 (0.991, 1.005)	0.604
*Genus*	*Howardella*	10	MR Egger	0.003	1.006 (0.999, 1.012)	0.152
			Weighted median	0.001	1.001 (0.999, 1.003)	0.506
			Inverse variance weighted	0.001	1.002 (1.000, 1.003)	0.033
			Simple mode	0.002	1.000 (0.997, 1.003)	0.812
			Weighted mode	0.002	1.000 (0.997, 1.004)	0.809
*Genus*	*Christensenellaceae R.7 group*	10	MR Egger	0.005	0.993 (0.983, 1.004)	0.233
			Weighted median	0.002	0.996 (0.991, 1.000)	0.049
			Inverse variance weighted	0.002	0.996 (0.993, 1.000)	0.028
			Simple mode	0.004	0.993 (0.985, 1.001)	0.112
			Weighted mode	0.004	0.993 (0.985, 1.001)	0.131
*Genus*	*Eubacterium oxidoreducens group*	5	MR Egger	0.005	1.000 (0.991, 1.010)	0.924
			Weighted median	0.002	1.001 (0.998, 1.005)	0.374
			Inverse variance weighted	0.001	1.003 (1.000, 1.005)	0.046
			Simple mode	0.002	1.001 (0.997, 1.005)	0.592
			Weighted mode	0.002	1.001 (0.998, 1.005)	0.558

SNP, single nucleotide polymorphism; MR, mendelian randomization; SE, standard error; 95% CI, 95% confidence interval.

#### 3.2.2. Causal effect of gut microbiota on fracture risk

**Figure 3:**
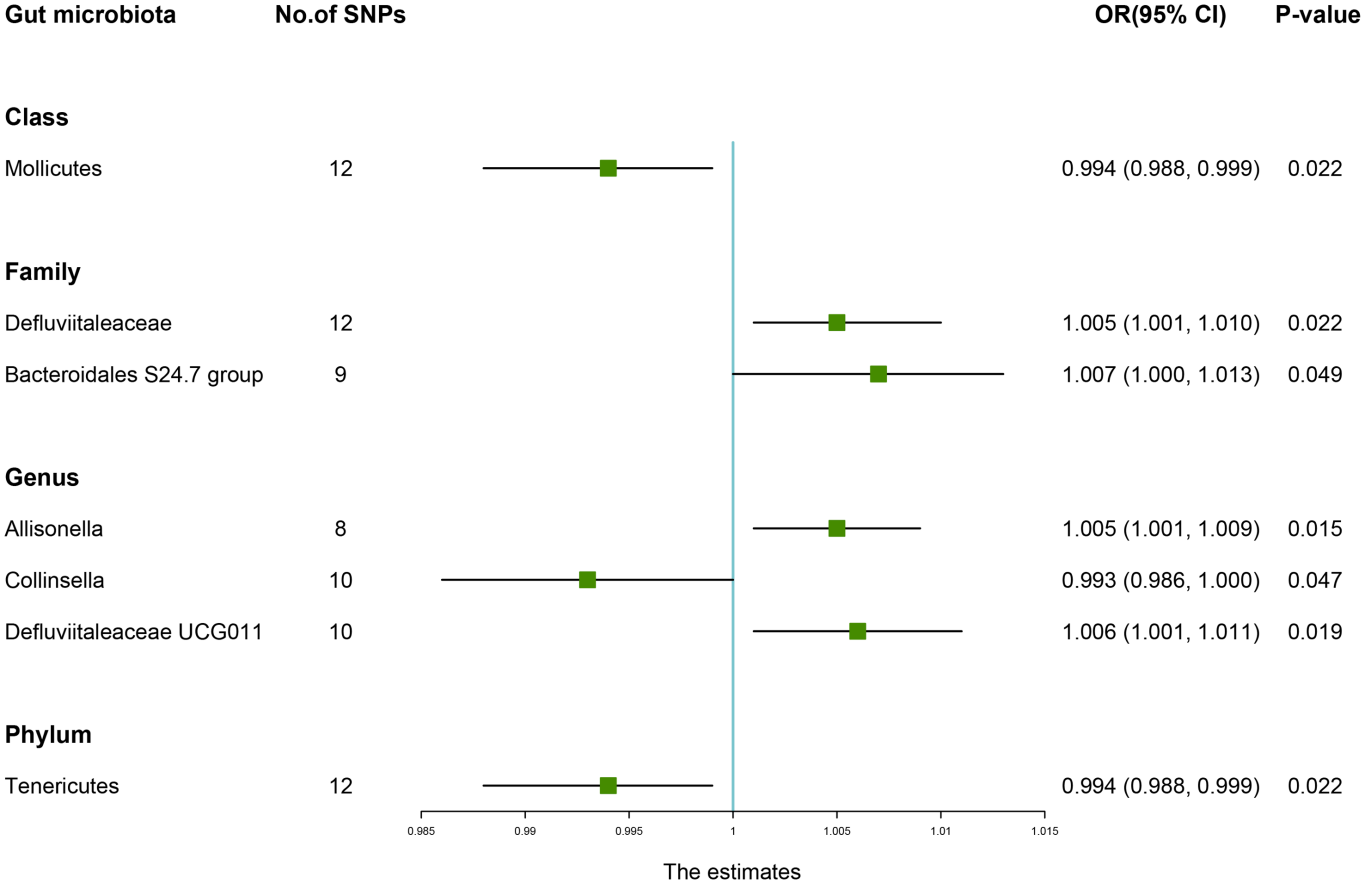
Forest plot of the causality between gut microbiota with the risk of fracture. The estimates: Inverse variance weighted (IVW) results of gut microbiota and fracture risk; *p-*value: *p-*value of the estimate. OR, odds ratio; SNP, single-nucleotide polymorphism.

The estimates of the IVW test showed that the genetically predicted four genera, namely, *Family Defluviitaleaceae* (OR: 1.005, 95% CI: 1.001–1.010, *p* = 0.022), *Family Bacteroidales S24.7 group* (OR: 1.007, 95% CI: 1.000–1.013, *p* = 0.049), *Genus Allisonella* (OR: 1.005, 95% CI: 1.001–1.009, *p* = 0.015), *Genus Defluviitaleaceae UCG011* (OR: 1.006, 95% CI: 1.001–1.011, *p* = 0.019) were positively associated with the risk of fracture (Table 2). And higher genetically predicted *Class Mollicutes* (OR: 0.994, 95% CI: 0.988–0.999, *p* = 0.022), *Genus Collinsella* (OR: 0.993, 95% CI: 0.986–1.000, *p* = 0.047), and *Phylum Tenericutes* (OR: 0.994, 95% CI: 0.988–0.999, *p* = 0.022) were linked with a decreased fracture risk (Figure 3).

**Table 2:** Mendelian randomisation (MR) results of causal effects between gut microbiome and the risk of fracture.

Group	Bacterial traits	Nsnp	Methods	SE	OR (95% CI)	*p-*value
*Class*	*Mollicutes*	12	MR Egger	0.009	0.992 (0.974, 1.010)	0.400
			Weighted median	0.004	0.994 (0.986, 1.002)	0.140
			Inverse variance weighted	0.003	0.994 (0.988, 0.999)	0.022
			Simple mode	0.006	0.993 (0.981, 1.005)	0.301
			Weighted mode	0.006	0.995 (0.983, 1.006)	0.374
*Family*	*Defluviitaleaceae*	12	MR Egger	0.008	1.006 (0.990, 1.021)	0.494
			Weighted median	0.003	1.005 (0.998, 1.011)	0.149
			Inverse variance weighted	0.002	1.005 (1.001, 1.010)	0.022
			Simple mode	0.005	1.006 (0.995, 1.016)	0.324
			Weighted mode	0.005	1.005 (0.995, 1.015)	0.359
*Family*	*Bacteroidales S24.7 group*	9	MR Egger	0.016	1.019 (0.988, 1.051)	0.277
			Weighted median	0.004	1.004 (0.997, 1.011)	0.245
			Inverse variance weighted	0.003	1.007 (1.000, 1.013)	0.049
			Simple mode	0.006	1.003 (0.992, 1.015)	0.554
			Weighted mode	0.005	1.002 (0.991, 1.012)	0.776
*Genus*	*Allisonella*	8	MR Egger	0.014	1.007 (0.980, 1.036)	0.613
			Weighted median	0.002	1.003 (0.998, 1.008)	0.272
			Inverse variance weighted	0.002	1.005 (1.001, 1.009)	0.015
			Simple mode	0.004	1.002 (0.995, 1.009)	0.597
			Weighted mode	0.004	1.002 (0.995, 1.009)	0.562
*Genus*	*Collinsella*	10	MR Egger	0.014	0.994 (0.967, 1.021)	0.656
			Weighted median	0.005	0.994 (0.985, 1.003)	0.169
			Inverse variance weighted	0.004	0.993 (0.986, 1.000)	0.047
			Simple mode	0.007	0.994 (0.980, 1.008)	0.421
			Weighted mode	0.007	0.995 (0.981, 1.008)	0.477
*Genus*	*Defluviitaleaceae UCG011*	10	MR Egger	0.009	1.002 (0.984, 1.020)	0.838
			Weighted median	0.004	1.006 (0.999, 1.014)	0.070
			Inverse variance weighted	0.003	1.006 (1.001, 1.011)	0.019
			Simple mode	0.006	1.008 (0.995, 1.021)	0.239
			Weighted mode	0.006	1.006 (0.994, 1.018)	0.331
*Phylum*	*Tenericutes*	12	MR Egger	0.009	0.992 (0.974, 1.010)	0.400
			Weighted median	0.004	0.994 (0.987, 1.001)	0.114
			Inverse variance weighted	0.003	0.994 (0.988, 0.999)	0.022
			Simple mode	0.007	0.993 (0.981, 1.066)	0.331
			Weighted mode	0.006	0.995 (0.982, 1.007)	0.416

SNP, single nucleotide polymorphism; MR, mendelian randomization; SE, standard error; 95% CI, 95% confidence interval.

#### 3.2.3. Causal effect of gut microbiota on sarcopenia-related traits

**Figure 4:**
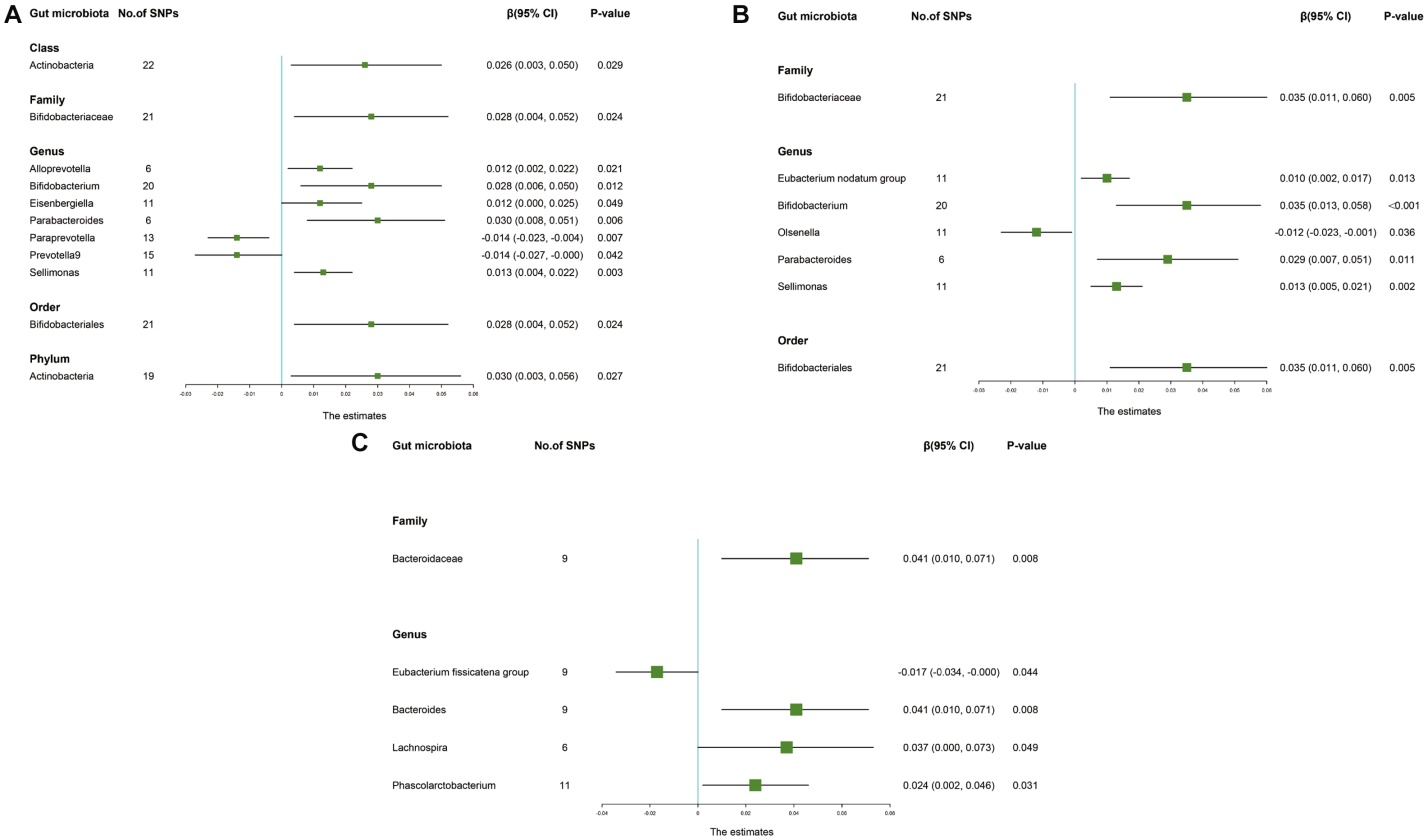
Forest plot of the causality between gut microbiota with sarcopenia-related traits. The estimates: Inverse variance weighted (IVW) results of gut microbiota and sarcopenia-related traits; *p-*value: *p-*value of the estimate. **(A)** Hand grip strength (Right); **(B)** Hand grip strength (Left); **(C)** Appendicular lean mass. SNP, single-nucleotide polymorphism.

The IVW results suggested that genetically predicted *Class Actinobacteria* (β: 0.026, 95% CI: 0.003–0.050, *p* = 0.029), *Family Bifidobacteriaceae* (β: 0.028, 95% CI: 0.004–0.052, *p* = 0.024), *Genus Alloprevotella* (β: 0.012, 95% CI: 0.002–0.022, *p* = 0.021), *Genus Bifidobacterium* (β: 0.028, 95% CI: 0.006–0.050, *p* = 0.012), *Genus Eisenbergiella* (β: 0.012, 95% CI: 0.000–0.025, *p* = 0.049), *Genus Parabacteroides* (β: 0.030, 95% CI: 0.008–0.051, *p* = 0.006), *Genus Sellimonas* (β: 0.013, 95% CI: 0.004–0.022, *p* = 0.003), *Order Bifidobacteriales* (β: 0.028, 95% CI: 0.004–0.052, *p* = 0.024), *Phylum Actinobacteria* (β: 0.030, 95% CI: 0.003–0.056, *p* = 0.027) were linked to an increase in right handgrip strength, while *Genus Paraprevotella* (β: -0.014, 95% CI: −0.023–-0.004, *p* = 0.007) and *Genus Prevotella9* (β: -0.014, 95% CI: −0.027–0.000, *p* = 0.042) were associated with an decrease in right handgrip strength (Table 3). Similarly, we found that genetically predicted *Family Bifidobacteriaceae* (β: 0.035, 95% CI: 0.011–0.060, *p* = 0.005), *Genus Eubacterium nodatum group* (β: 0.010, 95% CI: 0.002–0.017, *p* = 0.013), *Genus Bifidobacterium* (β: 0.035, 95% CI: 0.013–0.058, *P* <0.001), *Genus Parabacteroides* (β: 0.029, 95% CI: 0.007–0.051, *p* = 0.011), *Genus Sellimonas* (β: 0.013, 95% CI: 0.005–0.021, *p* = 0.002), *Order Bifidobacteriales* (β: 0.035, 95% CI: 0.011–0.060, *p* = 0.005) were also positively associated with left grip (Figures 4A,B). And the MR estimates of MR Egger and weighted median indicated that genetically predicted *Family Bifidobacteriaceae*, *Order Bifidobacteriales* were positively related to right and left handgrip strength (*P* <0.05), suggesting *Family Bifidobacteriaceae*, *Order Bifidobacteriales* were protective factors for sarcopenia (Table 4).

**Table 3:** Mendelian randomisation (MR) results of causal effects between gut microbiome and hand grip strength (Right).

Group	Bacterial traits	Nsnp	Methods	SE	β (95% CI)	*p-*value
*Class*	*Actinobacteria*	22	MR Egger	0.030	0.046 (−0.013, 0.106)	0.143
			Weighted median	0.010	0.013 (−0.006, 0.033)	0.179
			Inverse variance weighted	0.012	0.026 (0.003, 0.050)	0.029
			Simple mode	0.018	0.000 (−0.035, 0.034)	0.994
			Weighted mode	0.017	0.007 (−0.026, 0.040)	0.691
*Family*	*Bifidobacteriaceae*	21	MR Egger	0.034	0.146 (0.080, 0.213)	<0.001
			Weighted median	0.011	0.032 (0.010, 0.054)	0.004
			Inverse variance weighted	0.012	0.028 (0.004, 0.052)	0.024
			Simple mode	0.027	0.026 (−0.027, 0.080)	0.345
			Weighted mode	0.014	0.066 (0.037, 0.094)	<0.001
*Genus*	*Alloprevotella*	6	MR Egger	0.048	0.020 (−0.074, 0.115)	0.695
			Weighted median	0.007	0.015 (0.002, 0.028)	0.020
			Inverse variance weighted	0.005	0.012 (0.002, 0.022)	0.021
			Simple mode	0.009	0.015 (−0.003, 0.033)	0.156
			Weighted mode	0.009	0.015 (−0.002, 0.032)	0.141
*Genus*	*Bifidobacterium*	20	MR Egger	0.033	0.040 (−0.024, 0.104)	0.233
			Weighted median	0.011	0.031 (0.010, 0.051)	0.004
			Inverse variance weighted	0.011	0.028 (0.006, 0.050)	0.012
			Simple mode	0.024	0.021 (−0.026, 0.068)	0.393
			Weighted mode	0.021	0.069 (0.027, 0.111)	0.004
*Genus*	*Eisenbergiella*	11	MR Egger	0.051	0.043 (−0.057, 0.144)	0.423
			Weighted median	0.007	0.009 (−0.005, 0.023)	0.224
			Inverse variance weighted	0.006	0.012 (0.000, 0.025)	0.049
			Simple mode	0.011	0.009 (−0.012, 0.030)	0.438
			Weighted mode	0.011	0.009 (−0.013, 0.030)	0.452
*Genus*	*Parabacteroides*	6	MR Egger	0.033	0.012 (−0.053, 0.076)	0.741
			Weighted median	0.014	0.028 (0.000, 0.056)	0.047
			Inverse variance weighted	0.011	0.030 (0.008, 0.051)	0.006
			Simple mode	0.019	0.034 (−0.003, 0.071)	0.136
			Weighted mode	0.018	0.019 (−0.015, 0.054)	0.326
*Genus*	*Paraprevotella*	13	MR Egger	0.016	−0.015 (−0.048, 0.017)	0.369
			Weighted median	0.007	−0.017 (−0.031, −0.004)	0.013
			Inverse variance weighted	0.005	−0.014 (−0.023, −0.004)	0.007
			Simple mode	0.011	−0.017 (−0.040, 0.005)	0.152
			Weighted mode	0.012	−0.018 (−0.040, 0.005)	0.157
*Genus*	*Prevotella9*	15	MR Egger	0.020	−0.029 (−0.068, 0.011)	0.177
			Weighted median	0.008	−0.016 (−0.032, 0.001)	0.062
			Inverse variance weighted	0.007	−0.014 (−0.027, −0.000)	0.042
			Simple mode	0.016	−0.031 (−0.063, 0.001)	0.077
			Weighted mode	0.017	−0.031 (−0.065, 0.003)	0.096
*Genus*	*Sellimonas*	11	MR Egger	0.020	0.036 (−0.003, 0.076)	0.107
			Weighted median	0.006	0.012 (0.001, 0.023)	0.029
			Inverse variance weighted	0.004	0.013 (0.004, 0.022)	0.003
			Simple mode	0.009	0.015 (−0.003, 0.032)	0.127
			Weighted mode	0.009	0.014 (−0.004, 0.032)	0.159
*Order*	*Bifidobacteriales*	21	MR Egger	0.034	0.146 (0.080, 0.213)	<0.001
			Weighted median	0.011	0.032 (0.010, 0.054)	0.004
			Inverse variance weighted	0.012	0.028 (0.004, 0.052)	0.024
			Simple mode	0.026	0.026 (−0.025, 0.078)	0.330
			Weighted mode	0.015	0.066 (0.036, 0.096)	<0.001
*Phylum*	*Actinobacteria*	19	MR Egger	0.048	0.127 (0.033, 0.221)	0.017
			Weighted median	0.011	0.011 (−0.011, 0.034)	0.318
			Inverse variance weighted	0.014	0.030 (0.003, 0.056)	0.027
			Simple mode	0.018	0.002 (−0.033, 0.038)	0.904
			Weighted mode	0.017	0.002 (−0.031, 0.034)	0.928

SNP, single nucleotide polymorphism; MR, mendelian randomization; SE, standard error; 95% CI, 95% confidence interval.

**Table 4:** Mendelian randomisation (MR) results of causal effects between gut microbiome and hand grip strength (Left).

Group	Bacterial traits	Nsnp	Methods	SE	β (95% CI)	*p-*value
*Family*	*Bifidobacteriaceae*	21	MR Egger	0.034	0.159 (0.092, 0.226)	<0.001
			Weighted median	0.011	0.032 (0.009, 0.054)	0.005
			Inverse variance weighted	0.013	0.035 (0.011, 0.060)	0.005
			Simple mode	0.031	0.004 (−0.056, 0.065)	0.888
			Weighted mode	0.026	0.081 (0.030, 0.131)	0.005
*Genus*	*Eubacterium nodatum group*	11	MR Egger	0.017	0.012 (−0.022, 0.046)	0.518
			Weighted median	0.005	0.006 (−0.005, 0.016)	0.308
			Inverse variance weighted	0.004	0.010 (0.002, 0.017)	0.013
			Simple mode	0.009	0.002 (−0.016, 0.021)	0.803
			Weighted mode	0.010	0.002 (−0.017, 0.021)	0.830
*Genus*	*Bifidobacterium*	20	MR Egger	0.033	0.046 (−0.020, 0.111)	0.190
			Weighted median	0.011	0.031 (0.009, 0.053)	0.006
			Inverse variance weighted	0.011	0.035 (0.013, 0.058)	<0.001
			Simple mode	0.029	0.014 (−0.043, 0.072)	0.625
			Weighted mode	0.042	0.081 (−0.001, 0.162)	0.067
*Genus*	*Olsenella*	11	MR Egger	0.021	−0.034 (−0.076, 0.008)	0.142
			Weighted median	0.006	−0.014 (−0.025, −0.002)	0.019
			Inverse variance weighted	0.006	−0.012 (−0.023, −0.001)	0.036
			Simple mode	0.009	−0.013 (−0.031, 0.004)	0.164
			Weighted mode	0.009	−0.013 (−0.030, 0.004)	0.157
*Genus*	*Parabacteroides*	6	MR Egger	0.038	0.042 (−0.032, 0.116)	0.328
			Weighted median	0.015	0.027 (−0.001–0.056)	0.06
			Inverse variance weighted	0.011	0.029 (0.007, 0.051)	0.011
			Simple mode	0.022	0.030 (−0.014, 0.074)	0.235
			Weighted mode	0.019	0.028 (−0.009, 0.065)	0.204
*Genus*	*Sellimonas*	11	MR Egger	0.019	0.038 (0.001, 0.074)	0.073
			Weighted median	0.006	0.012 (0.001, 0.023)	0.035
			Inverse variance weighted	0.004	0.013 (0.005, 0.021)	0.002
			Simple mode	0.009	0.012 (−0.007, 0.030)	0.236
			Weighted mode	0.009	0.011 (−0.007, 0.029)	0.261
*Order*	*Bifidobacteriales*	21	MR Egger	0.034	0.159 (0.092, 0.226)	<0.001
			Weighted median	0.012	0.032 (0.009, 0.054)	0.006
			Inverse variance weighted	0.013	0.035 (0.011, 0.060)	0.005
			Simple mode	0.034	0.004 (−0.062, 0.071)	0.898
			Weighted mode	0.025	0.081 (0.031, 0.130)	0.005

SNP, single nucleotide polymorphism; MR, mendelian randomization; SE, standard error; 95% CI, 95% confidence interval.

In addition, the estimates of IVW indicated that genetically predicted *Family Bacteroidaceae* (β: 0.041, 95% CI: 0.010–0.071, *p* = 0.008), *Genus Bacteroides* (β: 0.041, 95% CI: 0.010–0.071, *p* = 0.008), *Genus Lachnospira* (β: 0.037, 95% CI: 0.000–0.073, *p* = 0.049), *Genus Phascolarctobacterium* (β: 0.024, 95% CI: 0.002–0.046, *p* = 0.031) were positively correlated with ALM (Table 5). And genetically predicted *Genus Eubacterium fissicatena group* (β: -0.017, 95% CI: −0.034–-0.000, *p* = 0.044), which suggested that a 1-SD increase in genetically determined *Genus Eubacterium fissicatena group* was correlated with an increase in ALM by 0.017 g (Figure 4C).

**Table 5:** Mendelian randomisation (MR) results of causal effects between gut microbiome and appendicular lean mass.

Group	Bacterial traits	Nsnp	Methods	SE	β (95% CI)	*p-*value
*Family*	*Bacteroidaceae*	9	MR Egger	0.086	0.008 (−0.160, 0.176)	0.927
			Weighted median	0.017	0.022 (−0.012, 0.056)	0.197
			Inverse variance weighted	0.015	0.041 (0.010, 0.071)	0.008
			Simple mode	0.025	0.019 (−0.031, 0.069)	0.478
			Weighted mode	0.021	0.017 (−0.025, 0.058)	0.456
*Genus*	*Eubacterium fissicatena group*	9	MR Egger	0.047	−0.047 (−0.139, 0.046)	0.353
			Weighted median	0.009	−0.022 (−0.039, −0.006)	0.009
			Inverse variance weighted	0.008	−0.017 (−0.034, −0.000)	0.044
			Simple mode	0.013	−0.024 (−0.049, 0.002)	0.103
			Weighted mode	0.012	−0.023 (−0.047, 0.001)	0.095
*Genus*	*Bacteroides*	9	MR Egger	0.086	0.008 (−0.160, 0.176)	0.927
			Weighted median	0.018	0.022 (−0.013, 0.057)	0.215
			Inverse variance weighted	0.015	0.041 (0.010, 0.071)	0.008
			Simple mode	0.025	0.019 (−0.031, 0.068)	0.477
			Weighted mode	0.023	0.017 (−0.029, 0.062)	0.492
*Genus*	*Lachnospira*	6	MR Egger	0.098	0.186 (−0.007, 0.379)	0.132
			Weighted median	0.020	0.036 (−0.003, 0.075)	0.072
			Inverse variance weighted	0.019	0.037 (0.000, 0.073)	0.049
			Simple mode	0.028	0.033 (−0.022, 0.087)	0.290
			Weighted mode	0.027	0.032 (−0.020, 0.084)	0.281
*Genus*	*Phascolarctobacterium*	11	MR Egger	0.053	0.022 (−0.082, 0.125)	0.689
			Weighted median	0.013	0.014 (−0.011, 0.039)	0.279
			Inverse variance weighted	0.011	0.024 (0.002, 0.046)	0.031
			Simple mode	0.018	0.017 (−0.017, 0.052)	0.347
			Weighted mode	0.018	0.017 (−0.018, 0.052)	0.358

SNP, single nucleotide polymorphism; MR, mendelian randomization; SE, standard error; 95% CI, 95% confidence interval.

#### 3.2.4. Causal effect of gut microbiota on low back pain risk

**Figure 5:**
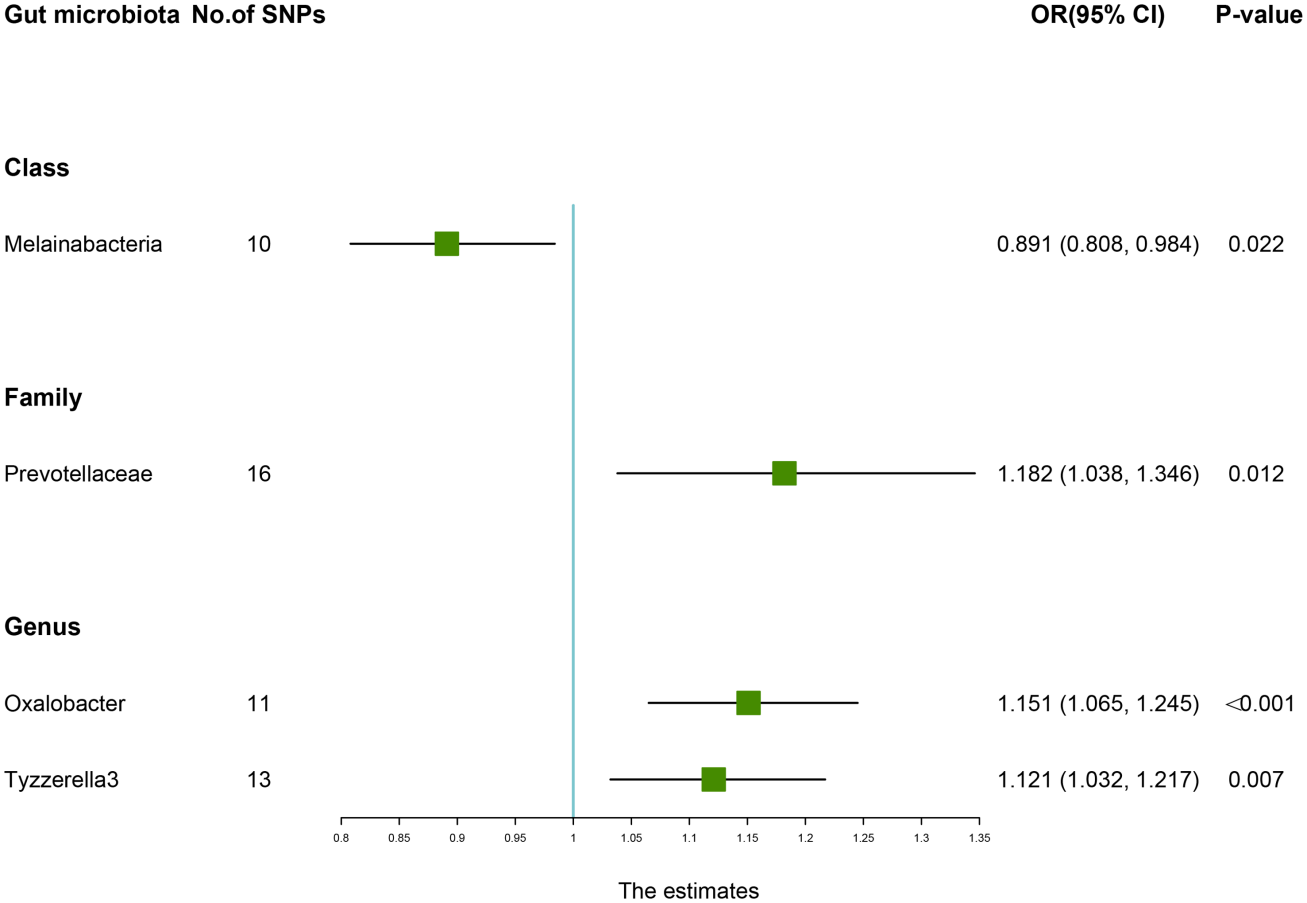
Forest plot of the causality between gut microbiota with the risk of low back pain. The estimates: Inverse variance weighted (IVW) results of gut microbiota and low back pain risk; *p-*value: *p-*value of the estimate. OR, odds ratio; SNP, single-nucleotide polymorphism.

As presented in Table 6, there was a causal correlation between four microbiotas and LBP. According to the results of the IVW method, higher genetically predicted *Family Prevotellaceae* (OR: 1.182, 95% CI: 1.038–1.346, *p* = 0.012), *Genus Oxalobacter* (OR: 1.151, 95% CI: 1.065–1.245, *P* <0.001), *Genus Tyzzerella3* (OR: 1.121, 95% CI: 1.032–1.217, *p* = 0.007) were correlated with a higher risk of LBP. However, *Class Melainabacteria* (OR: 0.891, 95% CI: 0.808–0.984, *p* = 0.022) was linked with a lower risk of LBP, which indicated that a 1-SD increase in *Class Melainabacteria* was correlated with a 10.9% decrease in LBP risk (Figure 5).

**Table 6:** Mendelian randomisation (MR) results of causal effects between gut microbiome and the risk of low back pain.

Group	Bacterial traits	Nsnp	Methods	SE	OR (95% CI)	*p-*value
*Class*	*Melainabacteria*	10	MR Egger	0.161	0.848 (0.618, 1.162)	0.334
			Weighted median	0.066	0.934 (0.820, 1.063)	0.301
			Inverse variance weighted	0.050	0.891 (0.808, 0.984)	0.022
			Simple mode	0.097	0.936 (0.774, 1.131)	0.509
			Weighted mode	0.085	0.927 (0.784, 1.096)	0.396
*Family*	*Prevotellaceae*	16	MR Egger	0.251	1.316 (0.804, 2.151)	0.293
			Weighted median	0.086	1.149 (0.970, 1.362)	0.107
			Inverse variance weighted	0.066	1.182 (1.038, 1.346)	0.012
			Simple mode	0.157	1.139 (0.837, 1.551)	0.421
			Weighted mode	0.154	1.125 (0.831, 1.522)	0.459
*Genus*	*Oxalobacter*	11	MR Egger	0.187	1.037 (0.719, 1.496)	0.848
			Weighted median	0.053	1.130 (1.018, 1.254)	0.022
			Inverse variance weighted	0.040	1.151 (1.065, 1.245)	<0.001
			Simple mode	0.099	1.050 (0.864, 1.276)	0.634
			Weighted mode	0.086	1.057 (0.893, 1.252)	0.534
*Genus*	*Tyzzerella3*	13	MR Egger	0.241	1.053 (0.656, 1.690)	0.834
			Weighted median	0.055	1.123 (1.009, 1.251)	0.035
			Inverse variance weighted	0.042	1.121 (1.032, 1.217)	0.007
			Simple mode	0.087	1.124 (0.948, 1.332)	0.203
			Weighted mode	0.086	1.128 (0.953, 1.336)	0.186

SNP, single nucleotide polymorphism; MR, mendelian randomization; SE, standard error; 95% CI, 95% confidence interval.

#### 3.2.5. Causal effect of gut microbiota on rheumatoid arthritis risk

**Figure 6:**
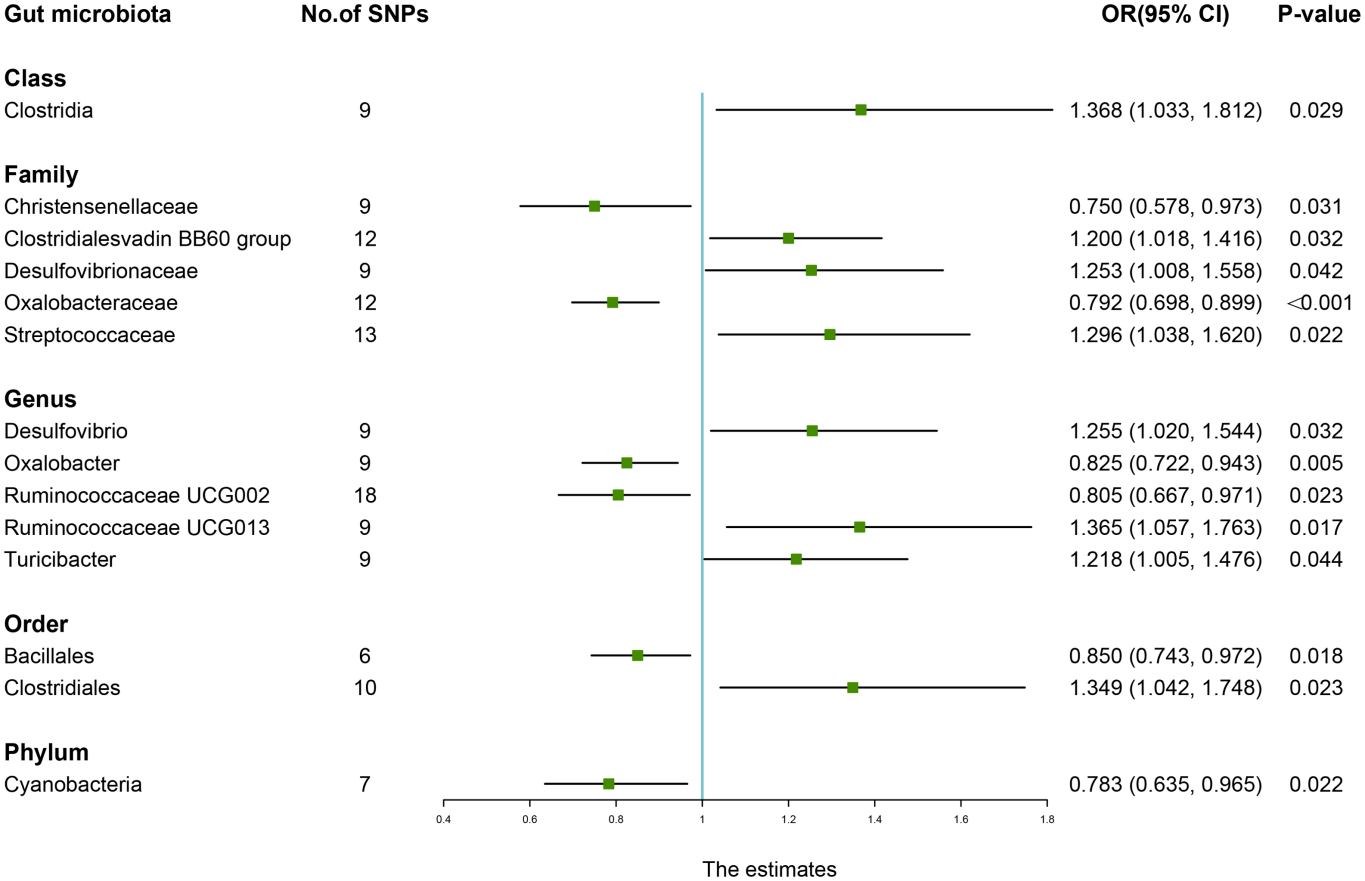
Forest plot of the causality between gut microbiota with the risk of rheumatoid arthritis. The estimates: Inverse variance weighted (IVW) results of gut microbiota and rheumatoid arthritis risk; *p-*value: *p-*value of the estimate. OR, odds ratio; SNP, single-nucleotide polymorphism.

As shown in Table 7, the IVW results suggested that genetically predicted *Class Clostridia* (OR: 1.368, 95% CI: 1.033–1.812, *p* = 0.029), *Family Clostridialesvadin BB60 group* (OR: 1.200, 95% CI: 1.018–1.416, *p* = 0.032), *Family Desulfovibrionaceae* (OR: 1.253, 95% CI: 1.008–1.558, *p* = 0.042), *Family Streptococcaceae* (OR: 1.296, 95% CI: 1.038–1.620, *p* = 0.022), *Genus Desulfovibrio* (OR: 1.255, 95% CI: 1.020–1.544, *p* = 0.032), *Genus Ruminococcaceae UCG013* (OR: 1.365, 95% CI: 1.057–1.763, *p* = 0.017), *Genus Turicibacter* (OR: 1.218, 95% CI: 1.005–1.476, *p* = 0.044), and *Order Clostridiales* (OR: 1.349, 95% CI: 1.042–1.748, *p* = 0.023) were positively associated with the risk of RA. By contrast, there was evidence that increasing abundance of *Family Christensenellaceae* (OR: 0.750, 95% CI: 0.578–0.973, *p* = 0.031), *Family Oxalobacteraceae* (OR: 0.792, 95% CI: 0.698–0.899, *P* <0.001), *Genus Oxalobacter* (OR: 0.825, 95% CI: 0.722–0.943, *p* = 0.005), *Genus Ruminococcaceae UCG002* (OR: 0.805, 95% CI: 0.667–0.971, *p* = 0.023), *Order Bacillales* (OR: 0.850, 95% CI: 0.743–0.972, *p* = 0.018), and *Phylum Cyanobacteria* (OR: 0.783, 95% CI: 0.635–0.965, *p* = 0.022) have a protective effect on RA risk (Figure 6).

**Table 7:** Mendelian randomisation (MR) results of causal effects between gut microbiome and the risk of rheumatoid arthritis.

Group	Bacterial traits	Nsnp	Methods	SE	OR (95% CI)	*p-*value
*Class*	*Clostridia*	9	MR Egger	0.648	1.589 (0.447, 5.655)	0.497
			Weighted median	0.187	1.386 (0.960, 2.000)	0.092
			Inverse variance weighted	0.143	1.368 (1.033, 1.812)	0.029
			Simple mode	0.316	1.475 (0.793, 2.742)	0.231
			Weighted mode	0.293	1.420 (0.800, 2.521)	0.229
*Family*	*Christensenellaceae*	9	MR Egger	0.317	0.727 (0.390, 1.354)	0.348
			Weighted median	0.164	0.765 (0.554, 1.055)	0.104
			Inverse variance weighted	0.133	0.750 (0.578, 0.973)	0.031
			Simple mode	0.230	0.790 (0.503, 1.240)	0.293
			Weighted mode	0.213	0.772 (0.509, 1.171)	0.252
*Family*	*Clostridialesvadin BB60 group*	12	MR Egger	0.231	1.101 (0.700, 1.732)	0.715
			Weighted median	0.119	1.307 (1.036, 1.649)	0.020
			Inverse variance weighted	0.084	1.200 (1.018, 1.416)	0.032
			Simple mode	0.208	1.405 (0.934, 2.114)	0.139
			Weighted mode	0.159	1.400 (1.025, 1.914)	0.089
*Family*	*Desulfovibrionaceae*	9	MR Egger	0.280	1.202 (0.695, 2.080)	0.531
			Weighted median	0.146	1.302 (0.978, 1.734)	0.095
			Inverse variance weighted	0.111	1.253 (1.008, 1.558)	0.042
			Simple mode	0.239	1.270 (0.795, 2.028)	0.335
			Weighted mode	0.193	1.320 (0.905, 1.927)	0.162
*Family*	*Oxalobacteraceae*	12	MR Egger	0.313	0.782 (0.424, 1.444)	0.450
			Weighted median	0.086	0.774 (0.654, 0.915)	0.002
			Inverse variance weighted	0.065	0.792 (0.698, 0.899)	<0.001
			Simple mode	0.127	0.778 (0.607, 0.997)	0.059
			Weighted mode	0.125	0.776 (0.608, 0.991)	0.047
*Family*	*Streptococcaceae*	13	MR Egger	0.498	1.535 (0.578, 4.078)	0.408
			Weighted median	0.157	1.596 (1.173, 2.173)	0.003
			Inverse variance weighted	0.114	1.296 (1.038, 1.620)	0.022
			Simple mode	0.226	1.578 (1.012, 2.460)	0.071
			Weighted mode	0.222	1.597 (1.033, 2.470)	0.051
*Genus*	*Desulfovibrio*	9	MR Egger	0.304	1.066 (0.588, 1.935)	0.839
			Weighted median	0.135	1.101 (0.845, 1.433)	0.484
			Inverse variance weighted	0.106	1.255 (1.020, 1.544)	0.032
			Simple mode	0.236	1.122 (0.706, 1.781)	0.625
			Weighted mode	0.163	1.070 (0.777, 1.472)	0.688
*Genus*	*Oxalobacter*	9	MR Egger	0.370	0.897 (0.435, 1.850)	0.777
			Weighted median	0.093	0.800 (0.667, 0.959)	0.014
			Inverse variance weighted	0.068	0.825 (0.722, 0.943)	0.005
			Simple mode	0.137	0.874 (0.668, 1.143)	0.335
			Weighted mode	0.137	0.795 (0.608, 1.040)	0.111
*Genus*	*Ruminococcaceae UCG002*	18	MR Egger	0.366	0.326 (0.159, 0.667)	0.007
			Weighted median	0.133	0.802 (0.618, 1.040)	0.102
			Inverse variance weighted	0.096	0.805 (0.667, 0.971)	0.023
			Simple mode	0.251	0.706 (0.432, 1.154)	0.192
			Weighted mode	0.218	0.725 (0.473, 1.112)	0.145
*Genus*	*Ruminococcaceae UCG013*	9	MR Egger	0.556	1.485 (0.499, 4.418)	0.501
			Weighted median	0.174	1.529 (1.087, 2.150)	0.019
			Inverse variance weighted	0.130	1.365 (1.057, 1.763)	0.017
			Simple mode	0.243	1.557 (0.966, 2.510)	0.118
			Weighted mode	0.223	1.531 (0.990, 2.369)	0.097
*Genus*	*Turicibacter*	9	MR Egger	0.519	1.224 (0.442, 3.385)	0.709
			Weighted median	0.133	1.144 (0.882, 1.483)	0.322
			Inverse variance weighted	0.098	1.218 (1.005, 1.476)	0.044
			Simple mode	0.220	1.093 (0.710, 1.681)	0.677
			Weighted mode	0.197	1.103 (0.750, 1.623)	0.614
*Order*	*Bacillales*	6	MR Egger	0.269	0.808 (0.476, 1.369)	0.472
			Weighted median	0.087	0.875 (0.737, 1.038)	0.139
			Inverse variance weighted	0.069	0.850 (0.743, 0.972)	0.018
			Simple mode	0.120	0.892 (0.705, 1.128)	0.383
			Weighted mode	0.103	0.900 (0.735, 1.101)	0.367
*Order*	*Clostridiales*	10	MR Egger	0.594	1.289 (0.402, 4.132)	0.680
			Weighted median	0.179	1.256 (0.885, 1.783)	0.215
			Inverse variance weighted	0.132	1.349 (1.042, 1.748)	0.023
			Simple mode	0.263	1.386 (0.829, 2.319)	0.244
			Weighted mode	0.233	1.309 (0.828, 2.067)	0.298
*Phylum*	*Cyanobacteria*	7	MR Egger	0.410	0.728 (0.326, 1.627)	0.474
			Weighted median	0.136	0.752 (0.576, 0.981)	0.028
			Inverse variance weighted	0.107	0.783 (0.635, 0.965)	0.022
			Simple mode	0.197	0.740 (0.502, 1.089)	0.164
			Weighted mode	0.182	0.746 (0.522, 1.064)	0.153

SNP, single nucleotide polymorphism; MR, mendelian randomization; SE, standard error; 95% CI, 95% confidence interval.

#### 3.2.6. Causal effect of gut microbiota on ankylosing spondylitis risk

**Figure 7:**
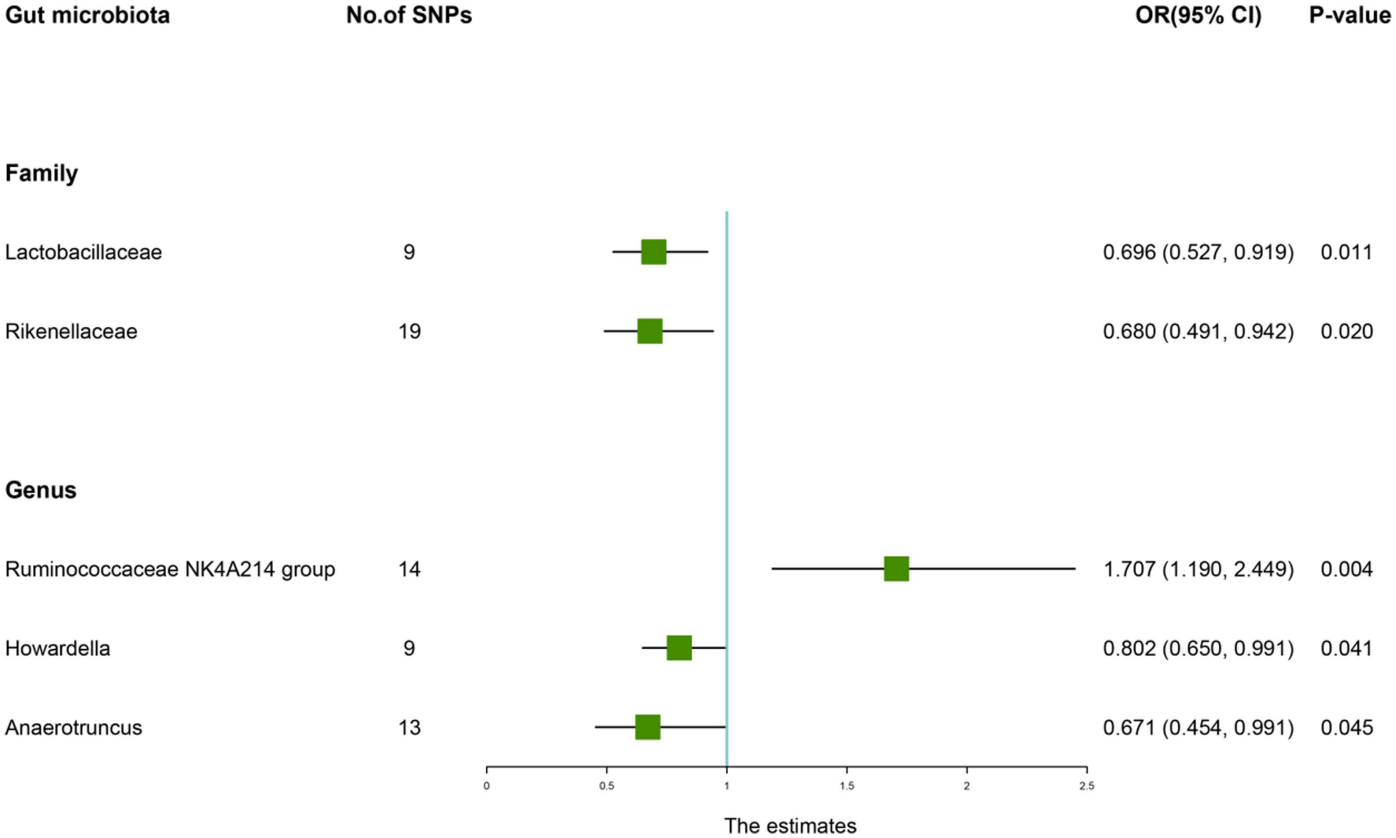
Forest plot of the causality between gut microbiota with the risk of ankylosing spondylitis. The estimates: Inverse variance weighted (IVW) results of gut microbiota and ankylosing spondylitis risk; *p-*value: *p-*value of the estimate. OR, odds ratio; SNP, single-nucleotide polymorphism.

According to the IVW method, genetically predicted *Family Lactobacillaceae* (OR: 0.696, 95% CI: 0.527–0.919, *p* = 0.011), *Family Rikenellaceae* (OR: 0.680, 95% CI: 0.491–0.942, *p* = 0.020), *Genus Howardella* (OR: 0.802, 95% CI: 0.650–0.991, *p* = 0.041), *Genus Anaerotruncus* (OR: 0.671, 95% CI: 0.454–0.991, *p* = 0.045) were negatively associated with the risk of AS (Table 8), while *Genus Ruminococcaceae NK4A214 group* (OR: 1.707, 95% CI: 1.190–2.449, *p* = 0.045) was positively linked with the AS risk (Figure 7).

**Table 8:** Mendelian randomisation (MR) results of causal effects between gut microbiome and the risk of ankylosing spondylitis.

Group	Bacterial traits	Nsnp	Methods	SE	OR (95% CI)	*p-*value
*Family*	*Lactobacillaceae*	9	MR Egger	0.350	0.615 (0.309, 1.221)	0.207
			Weighted median	0.193	0.783 (0.537, 1.143)	0.206
			Inverse variance weighted	0.142	0.696 (0.527, 0.919)	0.011
			Simple mode	0.302	0.527 (0.292, 0.952)	0.067
			Weighted mode	0.265	0.881 (0.523, 1.481)	0.644
*Family*	*Rikenellaceae*	19	MR Egger	0.524	0.766 (0.274, 2.137)	0.617
			Weighted median	0.224	0.600 (0.386, 0.931)	0.023
			Inverse variance weighted	0.166	0.680 (0.491, 0.942)	0.020
			Simple mode	0.409	0.546 (0.245, 1.216)	0.156
			Weighted mode	0.418	0.542 (0.239, 1.230)	0.160
*Genus*	*Ruminococcaceae NK4A214 group*	14	MR Egger	0.605	4.409 (1.347, 14.427)	0.030
			Weighted median	0.261	1.950 (1.168, 3.255)	0.011
			Inverse variance weighted	0.184	1.707 (1.190, 2.449)	0.004
			Simple mode	0.493	2.448 (0.931, 6.440)	0.093
			Weighted mode	0.411	2.448 (1.093, 5.484)	0.049
*Genus*	*Howardella*	9	MR Egger	0.428	0.615 (0.266, 1.424)	0.294
			Weighted median	0.157	0.882 (0.648, 1.200)	0.423
			Inverse variance weighted	0.108	0.802 (0.650, 0.991)	0.041
			Simple mode	0.257	0.964 (0.583, 1.595)	0.890
			Weighted mode	0.259	0.980 (0.590, 1.626)	0.939
*Genus*	*Anaerotruncus*	13	MR Egger	0.580	0.280 (0.090, 0.874)	0.051
			Weighted median	0.289	0.801 (0.454, 1.411)	0.442
			Inverse variance weighted	0.199	0.671 (0.454, 0.991)	0.045
			Simple mode	0.436	0.946 (0.402, 2.223)	0.900
			Weighted mode	0.426	0.900 (0.390, 2.074)	0.808

SNP, single nucleotide polymorphism; MR, mendelian randomization; SE, standard error; 95% CI, 95% confidence interval.

#### 3.2.7. Sensitivity analysis, Bonferroni-corrected test, and reverse analysis

We performed a series of sensitivity analyses to test the heterogeneity and horizontal pleiotropy of the selected IVs. Based on Cochran’s Q test, we observed no significant heterogeneity (*p* > 0.05) (Table 9). All *p* values of the MR-Egger intercept tests were > 0.05, which indicated no horizontal pleiotropy. Furthermore, we also did not discover any outliers through the MR-PRESSO global test (Table 9). Detailed scatter plots for each MR method analysis were shown in Supplementary Figures S2–S9. And results from a leave-one-out analysis demonstrated that no SNP was an influential outlier (Supplementary Figures S9–S16).

**Table 9:** Sensitivity analysis of the Mendelian randomization (MR) analysis results of gut microbiota and musculoskeletal diseases.

Outcome	Bacterial traits	Cochran Q statistic	Heterogeneity *p*-value	MR-Egger Intercept	Intercept *p*-value	MR-PRESSO Global test *p*-value
Osteoporosis	*NB1n*	4.318	0.987	0.000	0.413	0.987
	*Lachnospiraceae NK4A136 group*	11.362	0.581	0.000	0.086	0.514
	*Howardella*	5.752	0.765	−0.001	0.28	0.77
	*Christensenellaceae R.7 group*	10.056	0.3	0.000	0.55	0.36
	*Eubacterium oxidoreducens group*	4.423	0.3	0.00	0.673	0.41
Fracture	*Mollicutes*	10.467	0.48	0.00	0.848	0.50
	*Defluviitaleaceae*	13.793	0.245	0.00	0.573	0.26
	*Bacteroidales S24.7 group*	12.199	0.14	−0.0	0.4	0.18
	*Allisonella*	7.713	0.3	0.0	0.8	0.39
	*Collinsella*	3.987	0.91	0.00	0.9	0.92
	*Defluviitaleaceae UCG011*	8.947	0.44	0.00	0.6	0.46
	*Tenericutes*	10.467	0.489	0.000	0.848	0.521
Hand grip strength (Right)	*Actinobacteria*	43.759	0.119	0.002	0.360	0.209
	*Bifidobacteriaceae*	58.911	0.148	−0.009	0.177	0.451
	*Alloprevotella*	3.621	0.605	−0.001	0.871	0.645
	*Bifidobacterium*	48.382	0.268	−0.001	0.969	0.327
	*Eisenbergiella*	12.407	0.259	−0.004	0.372	0.280
	*Parabacteroides*	2.356	0.798	0.002	0.592	0.803
	*Paraprevotella*	7.685	0.809	0.001	0.721	0.809
	*Prevotella9*	21.374	0.092	0.002	0.441	0.098
	*Sellimonas*	8.671	0.371	−0.000	0.865	0.394
	*Actinobacteria*	12.803	0.119	0.002	0.702	0.308
Hand grip strength (Left)	*Bifidobacteriaceae*	60.348	0.799	−0.009	0.126	0.782
	*Eubacterium nodatum group*	9.233	0.510	−0.000	0.908	0.530
	*Bifidobacterium*	50.120	0.133	0.001	0.847	0.100
	*Olsenella*	18.383	0.488	0.003	0.305	0.066
	*Parabacteroides*	5.492	0.359	−0.001	0.728	0.446
	*Sellimonas*	6.242	0.620	0.000	0.990	0.651
	*Bifidobacteriales*	60.348	0.799	−0.009	0.126	0.762
Appendicular lean mass	*Bacteroidaceae*	12.005	0.151	0.002	0.711	0.195
	*Eubacterium fissicatena group*	16.318	0.380	0.004	0.540	0.517
	*Bacteroides*	12.005	0.151	0.002	0.711	0.175
	*Lachnospira*	7.805	0.167	−0.009	0.141	0.231
	*Phascolarctobacterium*	13.400	0.145	−0.001	0.825	0.174
Low back pain	*Melainabacteria*	9.168	0.422	−0.006	0.749	0.473
	*Prevotellaceae*	19.450	0.194	0.014	0.448	0.200
	*Oxalobacter*	6.227	0.796	−0.016	0.583	0.836
	*Tyzzerella3*	11.783	0.945	0.007	0.762	0.953
Rheumatoid arthritis	*Clostridia*	6.466	0.595	−0.010	0.819	0.649
	*Christensenellaceae*	0.309	1.000	0.003	0.916	1.000
	*Clostridialesvadin BB60 group*	8.826	0.638	0.008	0.698	0.653
	*Desulfovibrionaceae*	4.510	0.808	0.004	0.876	0.824
	*Oxalobacteraceae*	5.470	0.906	0.002	0.968	0.937
	*Streptococcaceae*	10.513	0.571	−0.013	0.734	0.600
	*Desulfovibrio*	10.225	0.250	0.017	0.583	0.287
	*Oxalobacter*	0.808	4.515	−0.012	0.825	0.819
	*Ruminococcaceae UCG002*	18.311	0.369	0.066	0.021	0.388
	*Ruminococcaceae UCG013*	12.877	0.116	−0.019	0.685	0.134
	*Turicibacter*	5.330	0.722	0.000	0.993	0.736
	*Bacillales*	2.280	0.809	0.007	0.855	0.840
	*Clostridiales*	6.184	0.721	0.003	0.939	0.760
	*Cyanobacteria*	6.867	0.333	0.009	0.862	0.422
Ankylosing spondylitis	*Lactobacillaceae*	4.146	0.844	0.016	0.710	0.843
	*Rikenellaceae*	10.762	0.904	−0.009	0.814	0.903
	*Ruminococcaceae NK4A214 group*	13.561	0.405	−0.073	0.126	0.443
	*Howardella*	6.444	0.598	0.042	0.542	0.543
	*Anaerotruncus*	10.645	0.560	0.063	0.138	0.582

MR-Egger, Mendelian randomization Egger.

According to the results of the Bonferroni-corrected test, *Genus Bifidobacterium* (β: 0.035, 95% CI: 0.013–0.058, *p* = 0.0002) was significantly correlated with left handgrip strength. In addition, higher level of *Genus Oxalobacter* retains a strong causal association with an increased risk of LBP (OR: 1.151, 95% CI: 1.065–1.245, *p* = 0.0003) and higher level of *Family Oxalobacteraceae* (OR: 0.792, 95% CI: 0.698–0.899, *p* = 0.0003) retains a strong causal association with a decreased risk of RA. In addition, reverse analysis demonstrates that fracture may result in a higher abundance of *Family Bacteroidales* (*p* = 0.030) and sarcopenia may lead to a higher abundance of *Genus Sellimonas* (*p* = 0.032) (Supplementary Table S10).

## 4. Discussion

To our knowledge, this was the first comprehensive and in-depth investigation of causal associations between gut microbiota and six common MSK diseases (OP, fracture, sarcopenia, LBP, RA, and AS) based on publicly available GWAS data. According to the findings of our study, a total of 57 gut microbiota were potentially causally associated with the progression of MSK diseases. After multiple testing corrections, we observed a significant causal relationship between *Genus Bifidobacterium*, *Genus Oxalobacter*, and *Family Oxalobacteraceae* with the risk of sarcopenia, LBP and RA, respectively. These findings might provide new ideas for the future treatment of MSK diseases by targeting the specific gut microbiota.

The gut microbiota consists of trillions of bacteria in the gastrointestinal tract, which have functions such as improving intestinal permeability, attenuating the inflammatory response, and participating in the immune regulation of the skeletal system (Rizzoli, 2019). In recent years, the “gut-bone/muscle” axis has received increasing attention in the field of bone health and orthopedic diseases and multiple studies indicated that gut microbiota composition is involved in the MSK disease pathogenesis through multiple pathways (Tu et al., 2021; Zhang et al., 2021). In our study, we discovered that genetically proxied higher abundance of *Genus Bifidobacterium* was significantly correlated with increased grip strength. And consistent directional effects for all analyses were observed in both MR Egger and weighted median methods, which suggests that *Bifidobacterium* might be a promising target for sarcopenia prevention. According to recent studies, *Bifidobacterium* has been identified as a critical taxon for frailty and sarcopenia in the elderly (Ticinesi et al., 2019). In a case–control study of elderly Chinese women, metagenomic sequencing of the gut microbiota revealed that the abundance of *Prevotella* and *Bifidobacterium* in the healthy control group was higher than those in the sarcopenia group (Wang et al., 2023). An animal experiment reported that *Bifidobacterium longum* could improve exercise-associated peripheral fatigue indicators (forelimb grip strength) and oxidative stress-related damage indicators in mice, which indicated *Bifidobacterium* may be involved in the occurrence and progression of age-related physical frailty and sarcopenia (Huang et al., 2020). In addition, a double-blind randomized clinical trial of older adults 65 aged 65 and over demonstrated that long-term intake of a prebiotic supplement containing *Bifidobacterium* significantly improved grip strength and reduced the level of fatigue (Buigues et al., 2016). *Bifidobacterium* might affect sarcopenia through its metabolites, such as short-chain fatty acids (acetate, propionate, and butyrate), which are important for immune regulation and metabolic homeostasis (Wang et al., 2015). *Bifidobacterium* also contributed to the absorption and utilization of vitamin D and minerals such as calcium, phosphorus, and iron, which are essential for muscle metabolism (Montazeri-Najafabady et al., 2019). Furthermore, *Bifidobacterium* was rich in genes encoding proteins related to the carbohydrate and amino acid transport and metabolism, influencing protein synthesis and nutrient utilization, which might also be one of the potential mechanisms by which *Bifidobacterium* prevents sarcopenia (Tu et al., 2023).

Our MR analysis also identified a causal relationship between *Genus Oxalobacter*, *Family Oxalobacteraceae* and the prevalence of LBP and RA, respectively. *Oxalobacter*, is a unique anaerobic bacterium that plays an important role in degrading dietary oxalate and stimulating oxalate secretion by the gut mucosa (Liu et al., 2017; Daniel et al., 2021). Recently, intensive studies have reported that *Oxalobacter* is closely related to the pathogenesis of kidney stones, hyperoxaluria, and chronic kidney disease (CKD) (Sidhu et al., 1999; Turkmen and Erdur, 2015; Falony, 2018). In addition, Li et al. observed that the diversity and relative abundance of gut microbiota in individuals with RA were significantly different from those in healthy controls. Specifically, the relative abundance of *Pelagibacterium* was higher and *Oxalobacter* and *Blautia* were lower in patients with RA (Li et al., 2021), which was consistent with our findings. Numerous studies emphasized the key role of the gut microbiota in RA pathogenesis, through a variety of mechanisms, including the production of proinflammatory metabolites, impairment of the intestinal mucosal barrier, and regulation of hormones throughout the body (Zhao et al., 2022). However, studies on the role of *Oxalobacter* in LBP are currently limited. Thus, it is necessary to further study the possible role of *Oxalobacter* in LBP and RA development.

In addition, we also observed some promising gut microbiota were associated with the risk of OP and AS. Several mechanisms seem to offer preliminary explanation for the relationship between gut flora and OP. On the one hand, the gut microbiota could regulate the Treg/Th17 cells balance or relevant cytokines through the immune system, affecting the intestinal and systemic immune states, thereby establishing a dynamic balance between osteoblasts (OB) and osteoclasts (OC), which is important for normal bone mass maintenance (Locantore et al., 2020). On the other hand, microbial metabolites such as SFAs, secondary bile acid (SBA), and indole derivatives participate in the reconstruction of bone resorption, metabolism and fracture healing by providing energy to gut epithelium cells and promoting calcium and phosphorus absorption (Tu et al., 2021). In addition, intestinal flora imbalance could promote the systemic chronic inflammatory response, which is closely related to the occurrence of AS (Guggino et al., 2021). Stebbings et al. demonstrated that the abundance of *Lachnospiraceae*, *Prevotellaceae*, and *Bacteroidaceae* was higher and that of *Ruminococcaceae* and *Rikenellaceae* was lower in AS patients compared with the healthy controls (Stebbings et al., 2002). According to an animal study in mice, regulating the gut microbiota and improving gut barrier function could delay AS progression (Liu et al., 2019). On the basis of the above evidence, we can further investigate how specific gut microbiota affect OP and AS.

Reverse MR analysis showed that fracture may lead to a higher abundance of *Family Bacteroidales* and sarcopenia may result in a lower abundance of *Genus Sellimonas*. According to a case–control study of 50 elderly patients, in patients with fracture, the relative abundance of *Bacteroidales* was higher and *Lachnospirales* was lower compared to healthy controls, suggesting that altering the microbiome may be an effective way to reduce fracture risk (Rosello-Anon et al., 2023). Another study of postmenopausal Japanese women reported that *Bacteroidels* and *Rikenellaceae* may be involved in bone metabolism and fracture risk (Ozaki et al., 2021). Furthermore, an animal study has also revealed that the proportion of *Firmicutes* and *Bacteroidetes* increased significantly by 16S RNA sequencing in postmenopausal osteoporosis mice (Wen et al., 2020). In addition, *Sellimonas* has been shown to be involved in restoring the balance of the intestinal flora (Munoz et al., 2020). Despite this, there has been a very limited amount of literature about *Sellimonas*. Thus, the underlying mechanism warrants future investigation.

Our study had several strengths. First of all, we used MR analysis to infer that the relationship between the gut microbiome and MSK diseases should be less susceptible to confounding and reverse causation than traditional observational analyses. Additionally, we analyzed the causal effect of each taxon on MSK diseases from the genus to the phylum level, which provides guidance for the prevention and treatment of MSK diseases by targeting specific gut microbiota in clinical practice. Third, the combination of stringent quality control procedures and multiple sensitivity analysis approaches enhances the credibility and robustness in the causal relationships inferred from the MR study. However, the present study also had some limitations that should be noted. Firstly, due to the lack of demographic data in the original study (e.g., gender, age, and race), we could not perform further subgroup analyses to obtain more specific effect relationships. Secondly, the GWAS data on gut microbiota used in this study are based on the population cohort from the largest macro-genome sequencing study to date. In the future, summary data of other gut microbiota needs to be obtained to provide a more comprehensive assessment of the causal relationship between gut microbes and the risk of MSK diseases. Last but not least, this study was confined to individuals of European origin and other populations require further MR studies since causal relationships may vary from race to race.

Overall, our study comprehensively evaluated the potential causal association between gut microbiota and six common MSK diseases. These findings provided new insights into the prevention, progression, and treatment of MSK diseases through targeting specific gut microbiota. In the future, more clinical trials and mechanism studies are needed to explore the exact mechanisms underlying the interactions between the gut microbiota and the prevalence of MSK diseases.

## 5. Conclusion

In summary, our study provided evidence of a potential causal relationship between specific gut microbiota and MSK diseases through the bi-directional MR analysis. These findings opened up new avenues for exploring the underlying mechanisms by which gut microbiota influence MSK diseases and contributed to the development of targeted interventions and personalized treatments.

## Data availability statement

The original contributions presented in the study are included in the article/Supplementary material, further inquiries can be directed to the corresponding authors.

## Author contributions

SC collected data. HH, XS, and GZ organized the study and performed the statistical analysis. QZ and ZL drafted the manuscript. All authors contributed to the article and approved the submitted version.

## Funding

This work was supported by the Elderly Health Research Project of Jiangsu Commission of Health (LKZ2022008), the Natural Science Foundation of Nanjing University of Chinese Medicine (XZR2021060), and the Foundation of The Second Affiliated Hospital of Nanjing University of Chinese Medicine (SEZ202003).

## Conflict of interest

The authors declare that the research was conducted in the absence of any commercial or financial relationships that could be construed as a potential conflict of interest.

## Publisher’s note

All claims expressed in this article are solely those of the authors and do not necessarily represent those of their affiliated organizations, or those of the publisher, the editors and the reviewers. Any product that may be evaluated in this article, or claim that may be made by its manufacturer, is not guaranteed or endorsed by the publisher.
