# Supplementary material for: Causal effects of specific gut microbiota on musculoskeletal diseases: a bidirectional two-sample Mendelian randomization study
Source: Front Microbiol. 2023 Aug 17;14:1238800. doi: 10.3389/fmicb.2023.1238800 (PMC10469765; doi:10.3389/fmicb.2023.1238800)
Supplement: Supplementary file 1 [file Data_Sheet_1.docx]

Supplementary Material

**Supplementary Figure S1.**

Scatter plots of causal estimates of exposure (Specific gut microbiota) on osteoporosis. Calculate the MR results of the remaining IVs after removing the IVs one by one. (A): *Order NB1n*; (B): *Genus LachnospiraceaeNK4A136group*; (C): *Genus Howardella*; (D): *Genus ChristensenellaceaeR.7group*; (E): *Genus Eubacteriumoxidoreducensgroup*; A
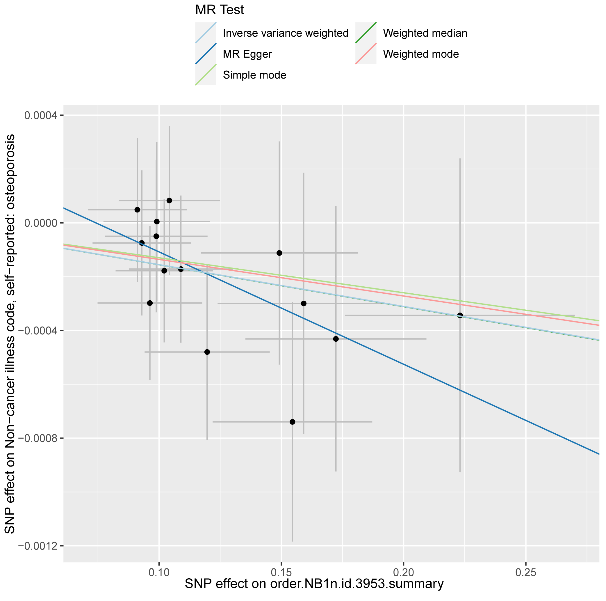
B
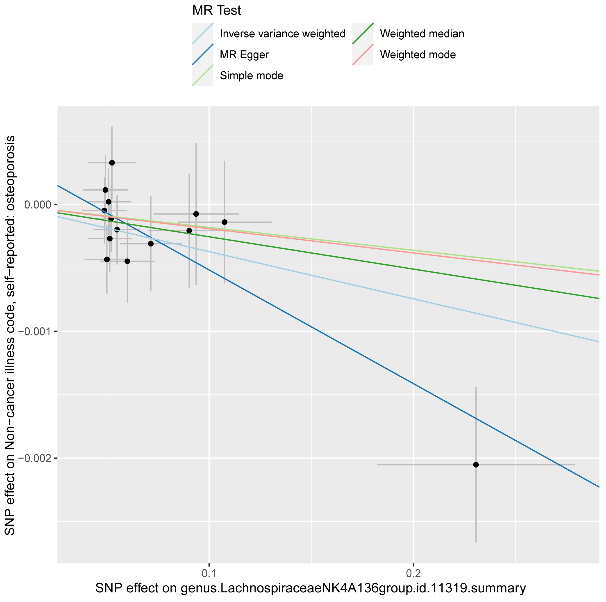


C
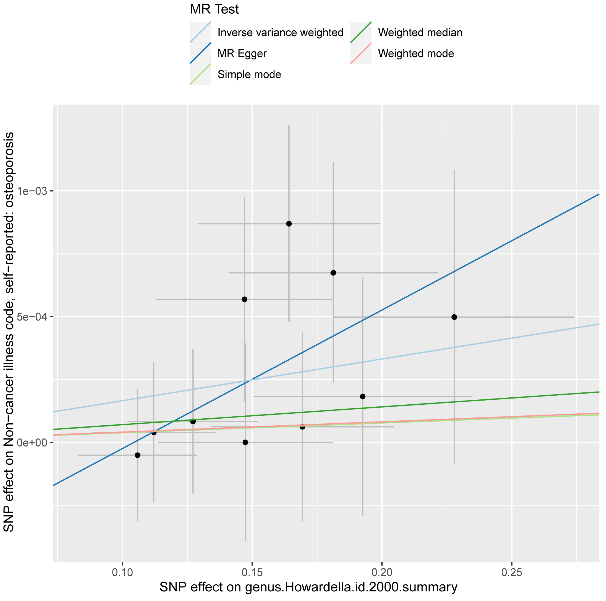
D
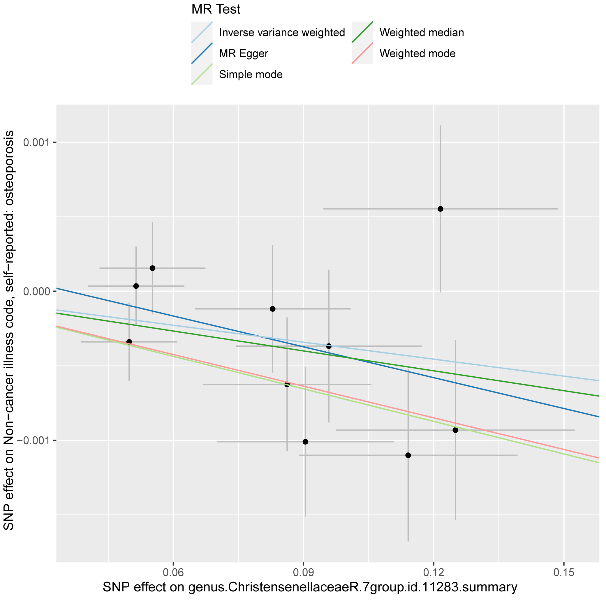


E
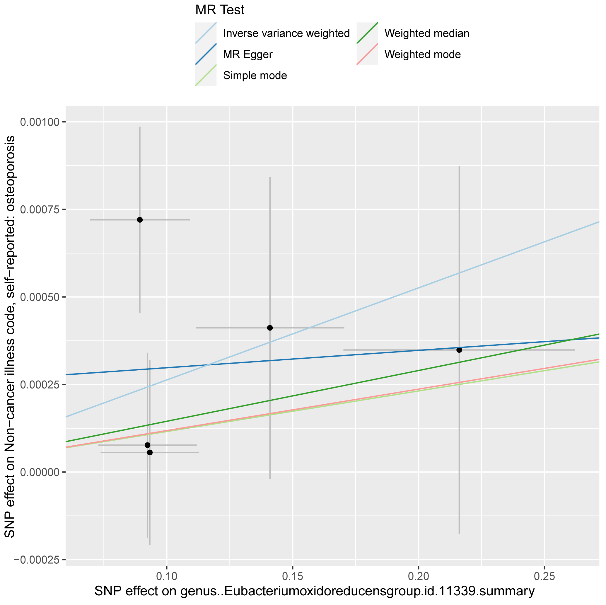


**Supplementary Figure S2.**

Scatter plots of causal estimates of exposure (Specific gut microbiota) on fracture. The slope of each line corresponding to the estimated MR effect in different models, including the conventional IVW, Weighted median, MR-Egger, Simple mode, and Weighted mode. (A): *Class Mollicutes*; (B): *Family Defluviitaleaceae*; (C): *Family BacteroidalesS24.7group*; (D): *Genus Allisonella*; (E): *Genus Collinsella*; (F): *Genus DefluviitaleaceaeUCG011*; (G): *Phylum Tenericutes*

A
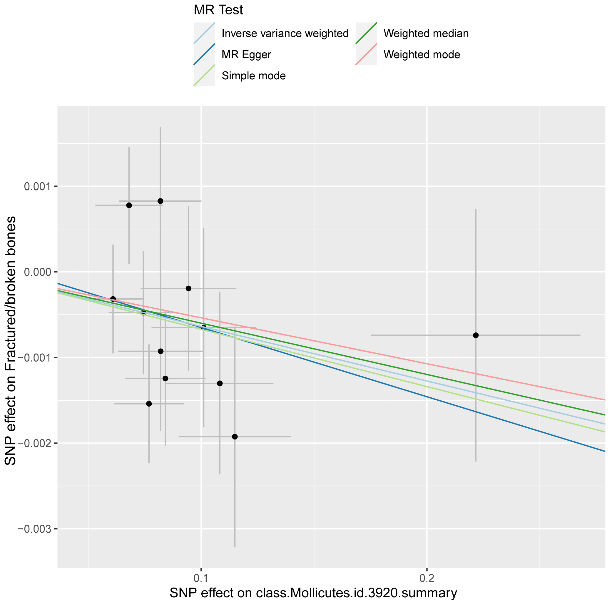
B
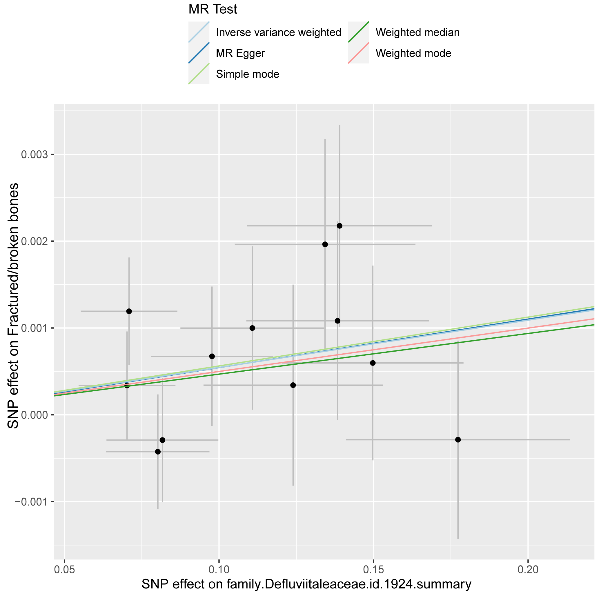


C
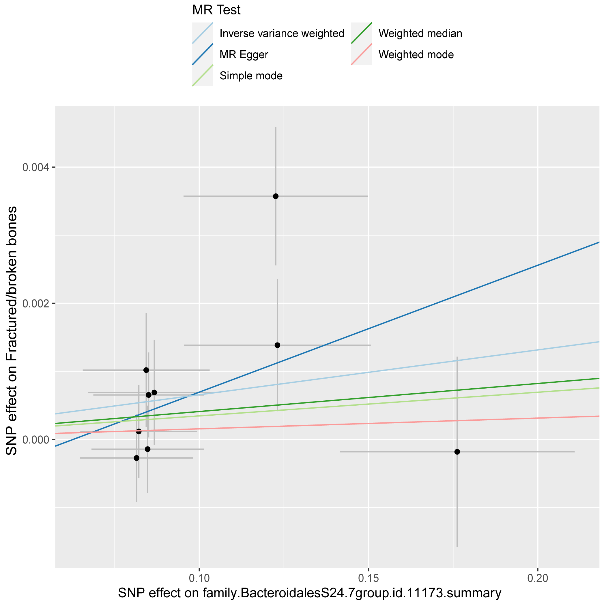
D
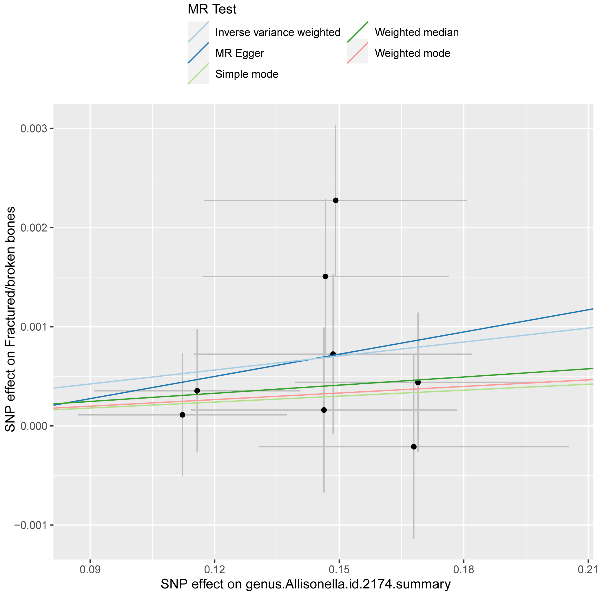


E
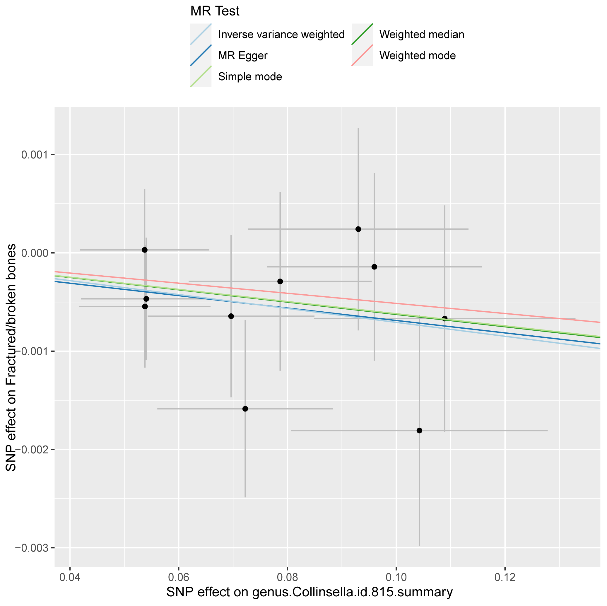
F
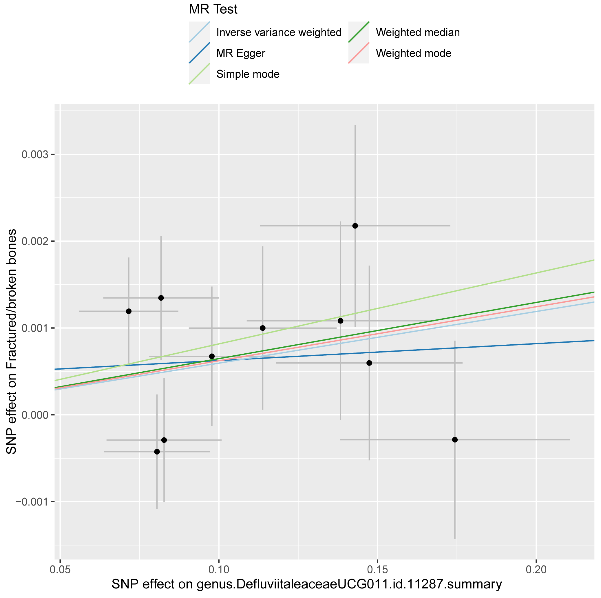


G
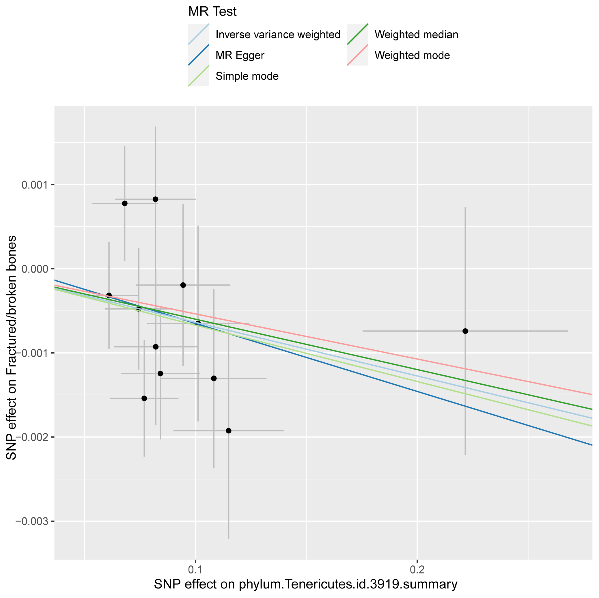


**Supplementary Figure S3.**

Scatter plots of causal estimates of exposure (Specific gut microbiota) on hand grip strength (Right). Calculate the MR results of the remaining IVs after removing the IVs one by one. (A): *Class Actinobacteria*; (B): *Family Bifidobacteriaceae*; (C): *Genus Alloprevotella*; (D): *Genus Bifidobacterium*; (E): *Genus Eisenbergiella*; (F): *Genus Parabacteroides;* (G): *Genus Paraprevotella*; (H): *Genus Prevotella9*; (I): *Genus Sellimonas*; (J): *Order Bifidobacteriales*; (K): *Phylum Actinobacteria*;

A
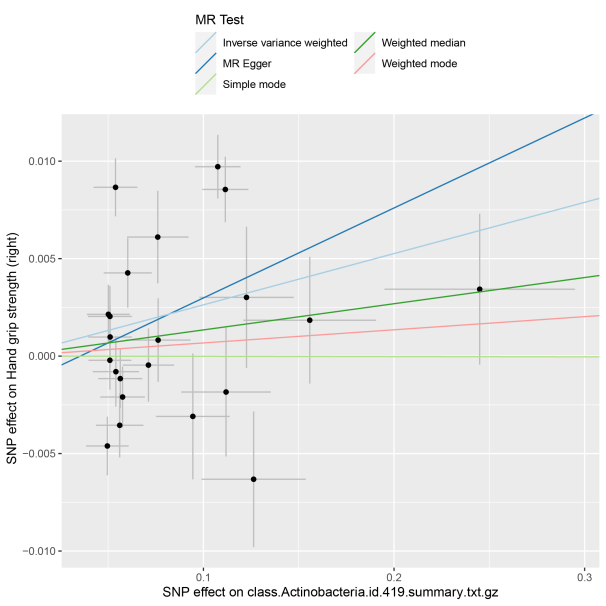
B
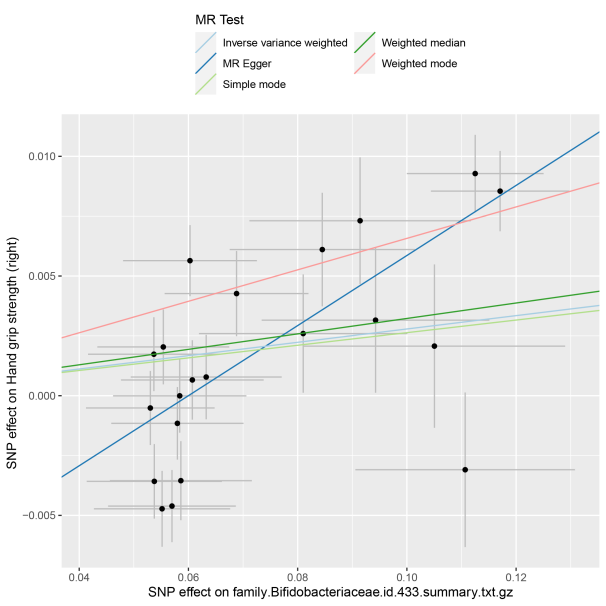


C
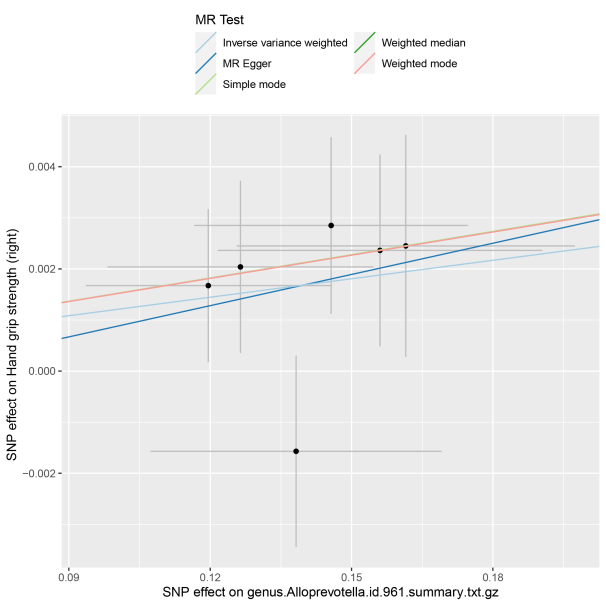
D
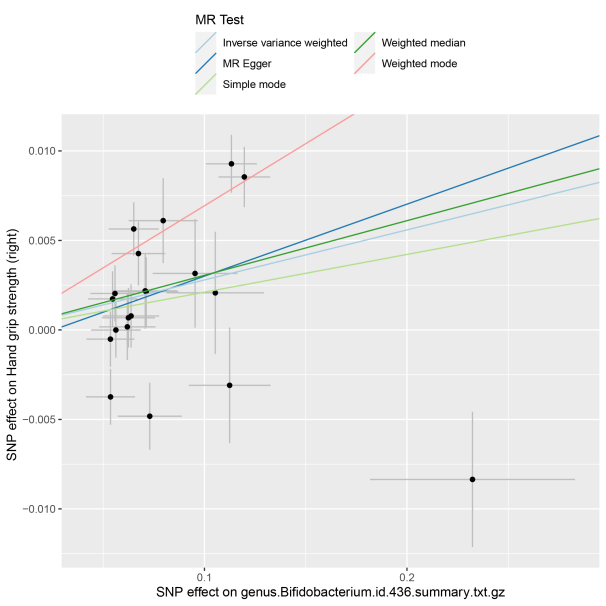


E
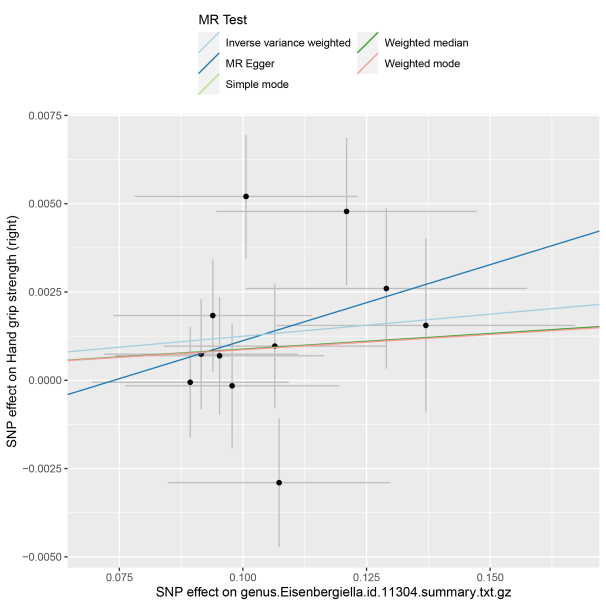
F
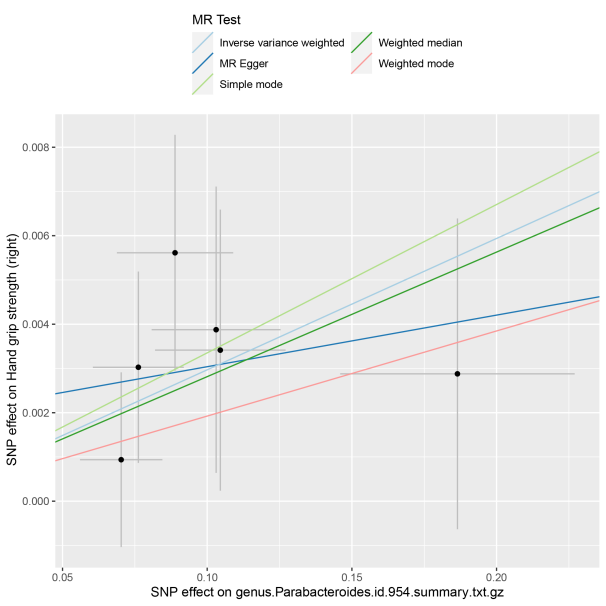


G
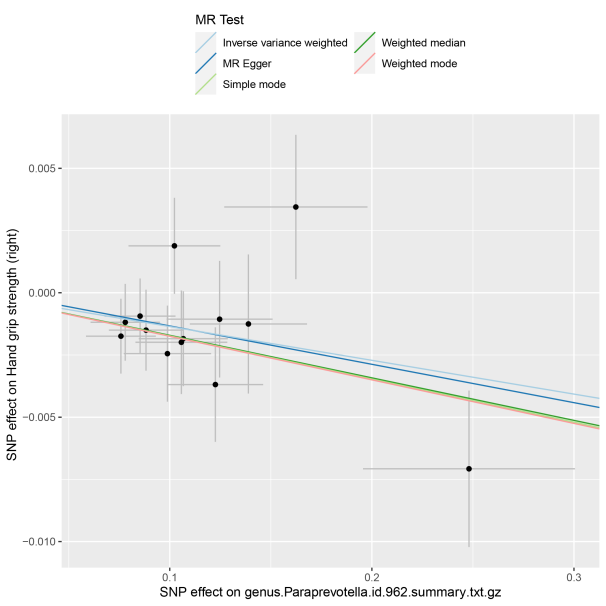
H
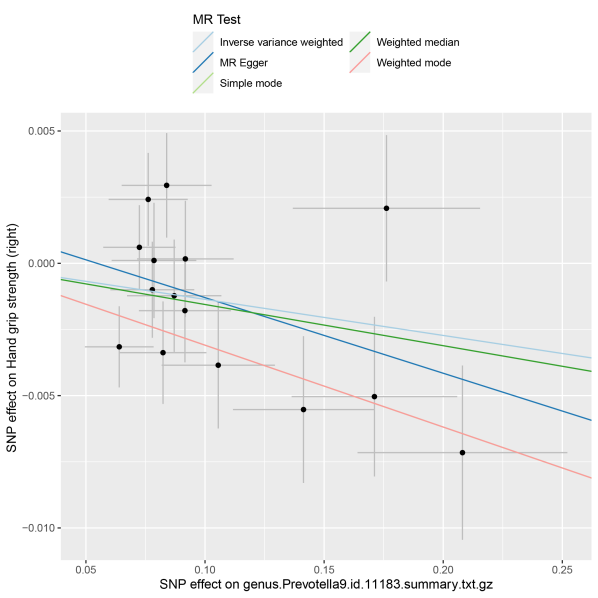


I
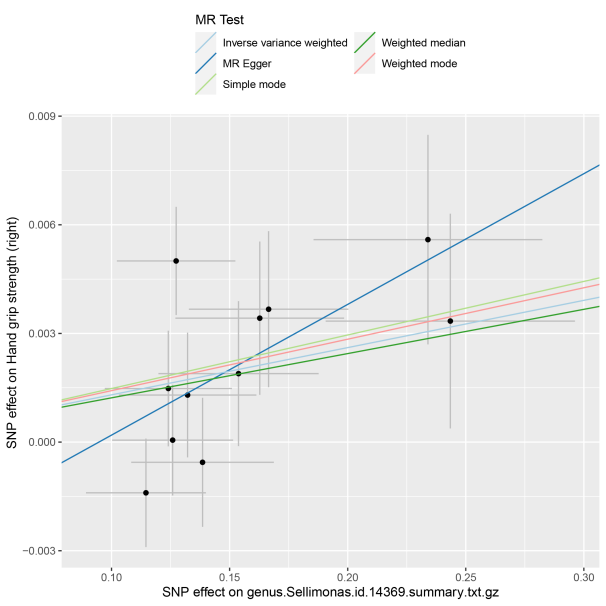
J
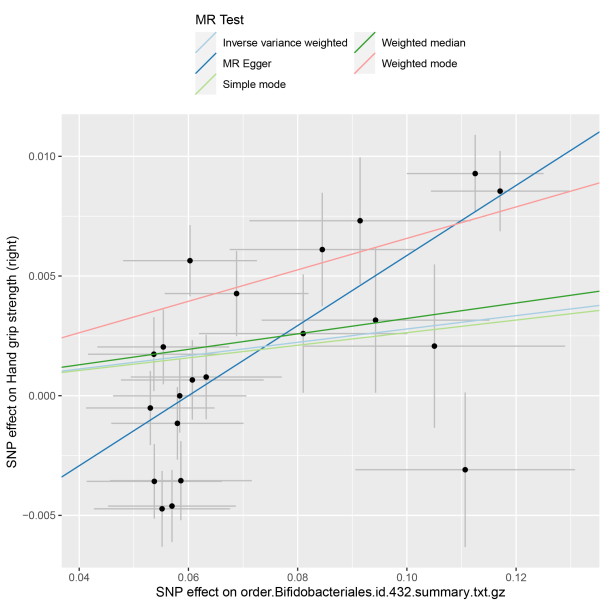


K
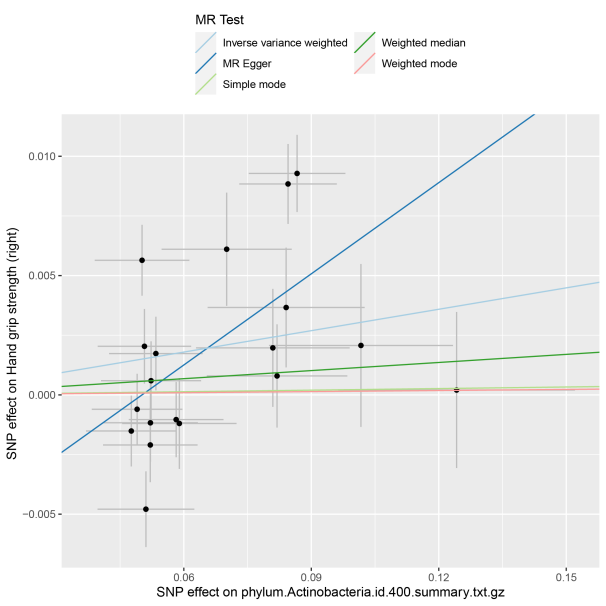


**Supplementary Figure S4.**

Scatter plots of causal estimates of exposure (Specific gut microbiota) on hand grip strength (Left). Calculate the MR results of the remaining IVs after removing the IVs one by one. (A): *Family Bifidobacteriaceae*; (B): *Genus Eubacterium nodatum group*; (C): *Genus Bifidobacterium*; (D): *Genus Olsenella*; (E): *Genus Parabacteroides*; (F): *Genus Sellimonas*; (G): *Order Bifidobacteriales*;

A
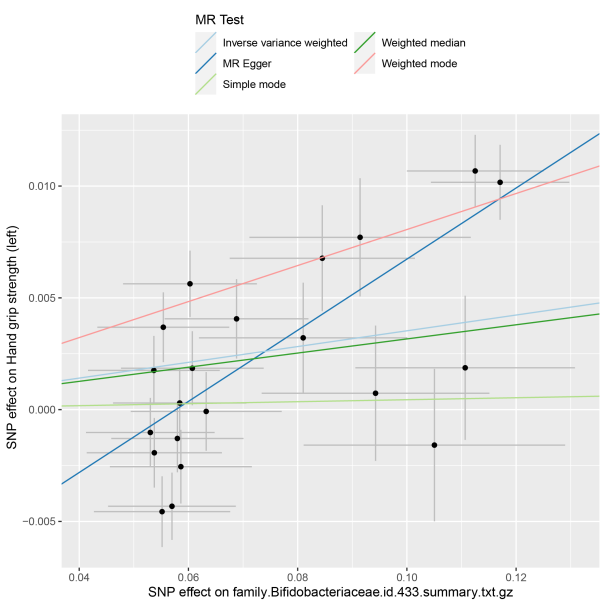
B
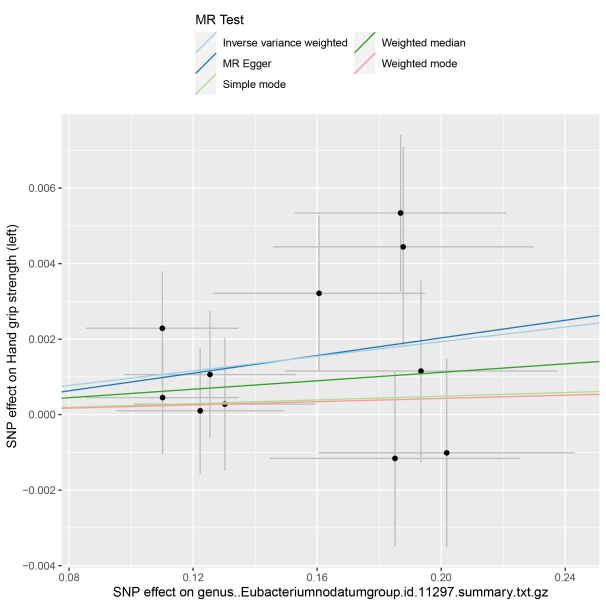


C
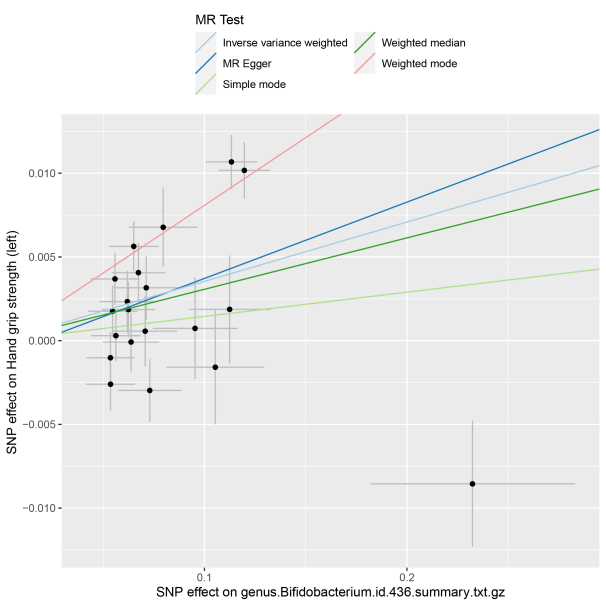
D
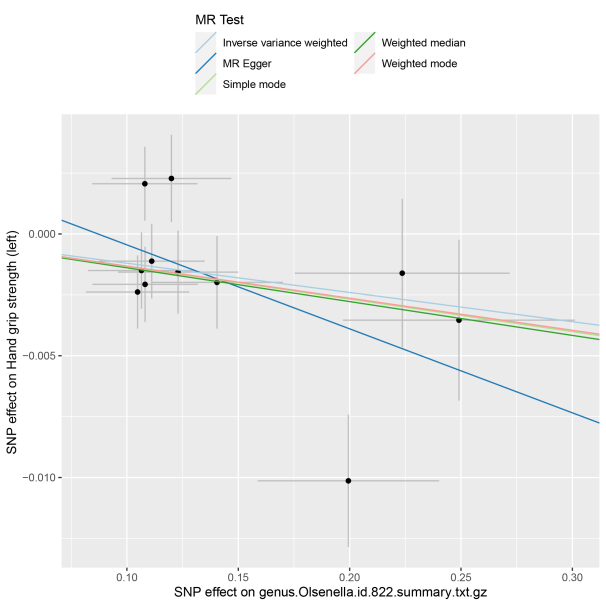


E
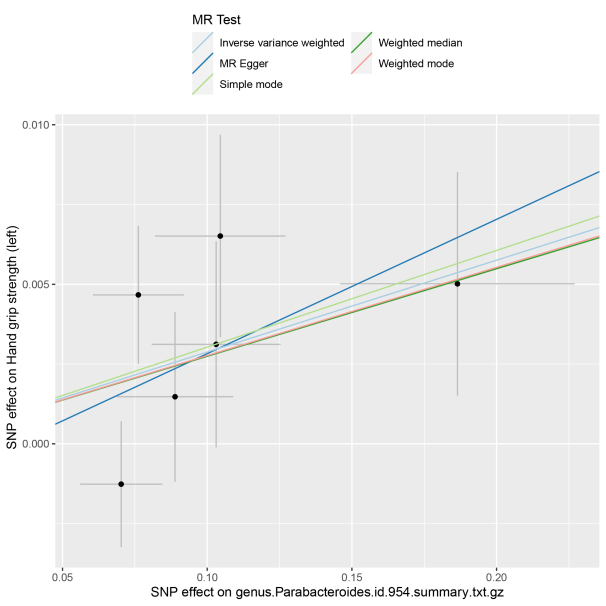
F
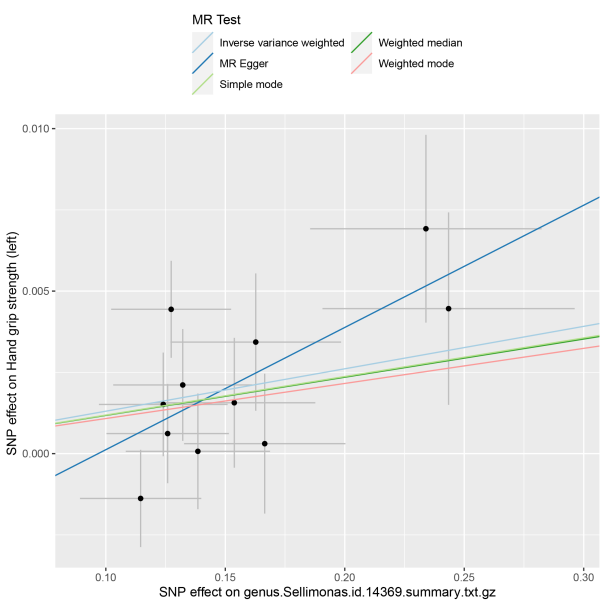


G
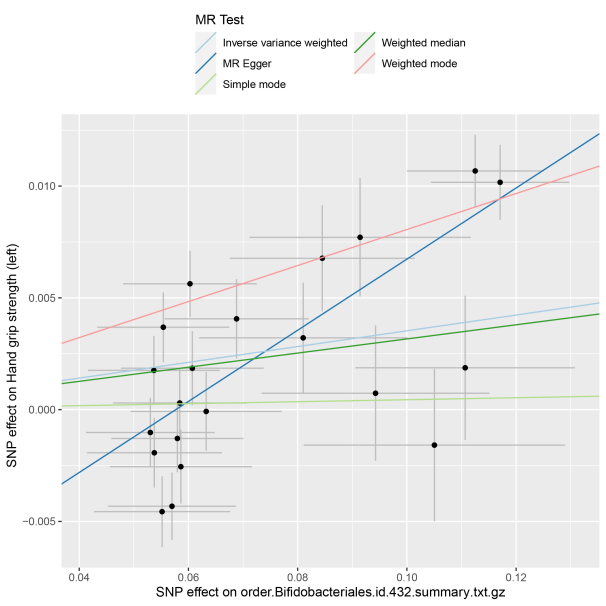


**Supplementary Figure S5.**

Scatter plots of causal estimates of exposure (Specific gut microbiota) on appendicular lean mass. Calculate the MR results of the remaining IVs after removing the IVs one by one. (A): *Family Bacteroidaceae*; (B): *Genus Eubacterium fissicatena group*; (C): *Genus Bacteroides*; (D): *Genus Lachnospira*; (E): *Genus Phascolarctobacterium*;

A
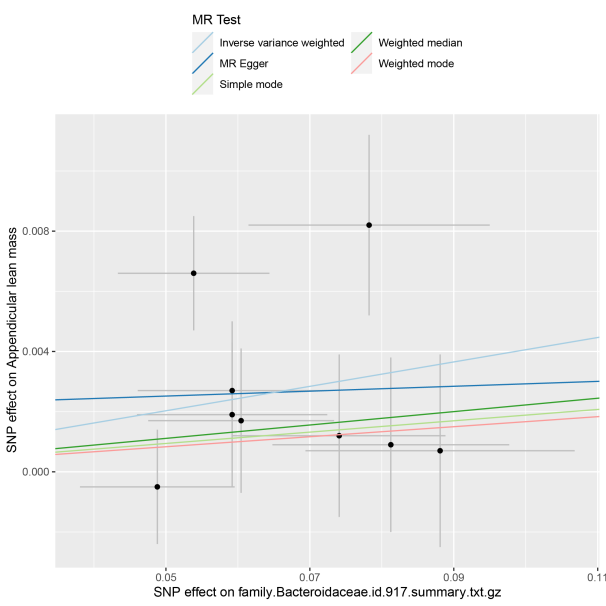
B
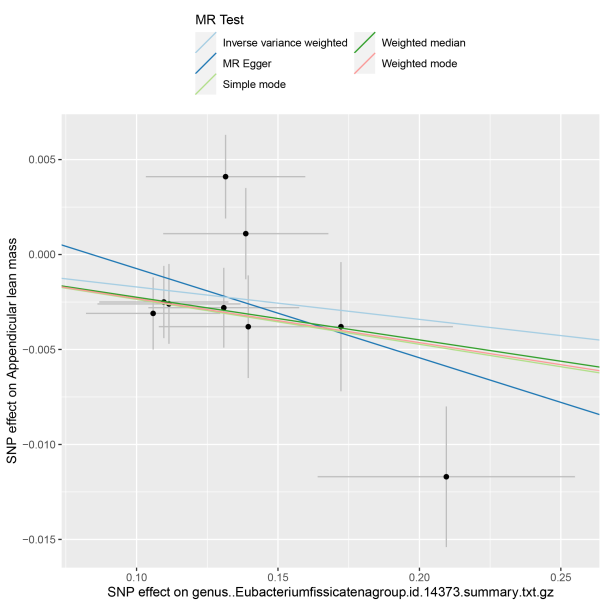


C
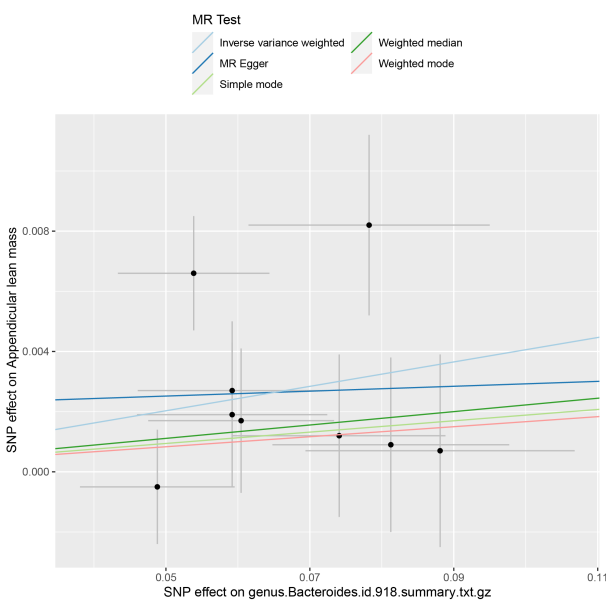
D
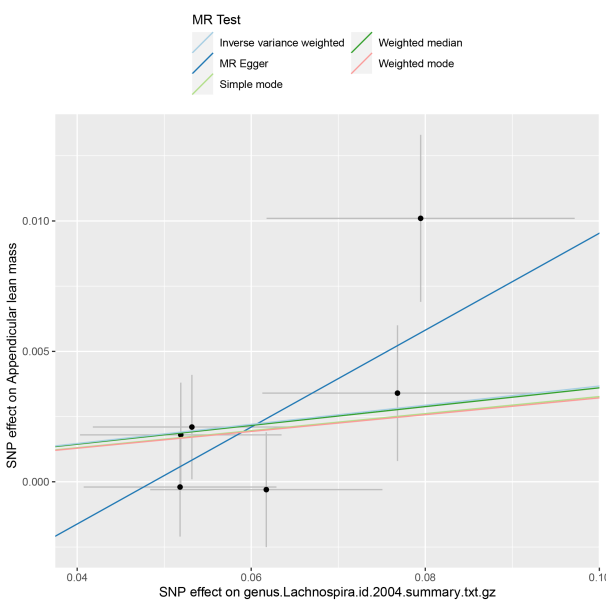


E
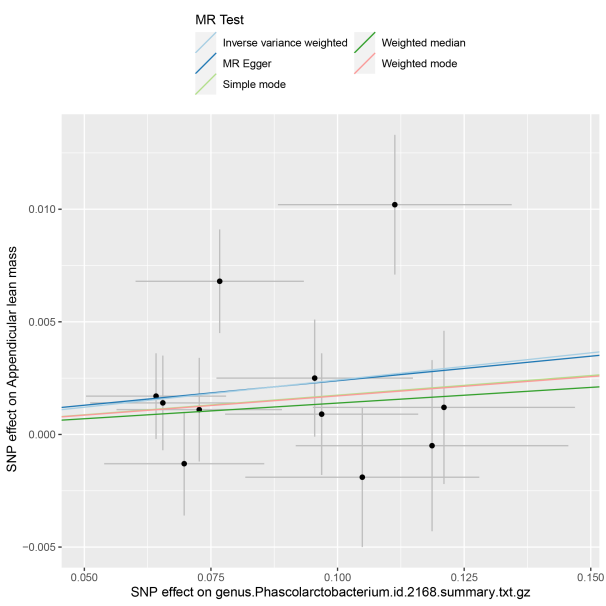


**Supplementary Figure S6.**

Scatter plots of causal estimates of exposure (Specific gut microbiota) on low back pain. The slope of each line corresponding to the estimated MR effect in different models, including the conventional IVW, Weighted median, MR-Egger, Simple mode, and Weighted mode. (A): *Class Melainabacteria*; (B): *Family Prevotellaceae*;(C): *Genus Oxalobacter*; (D): *Genus Tyzzerella3*

A**
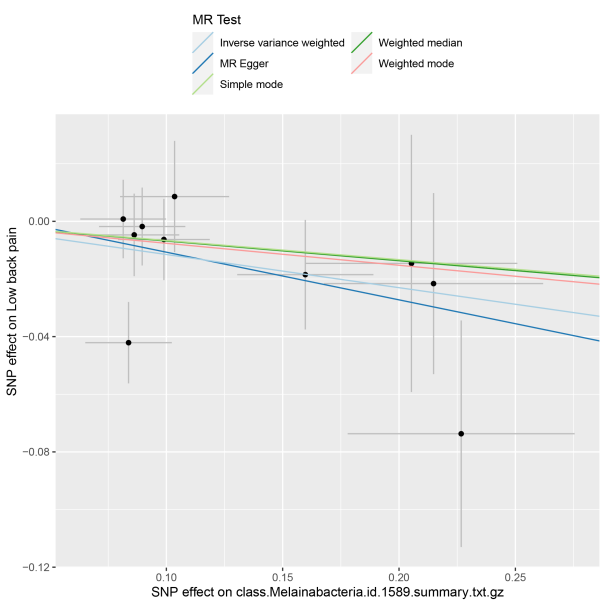
**B**
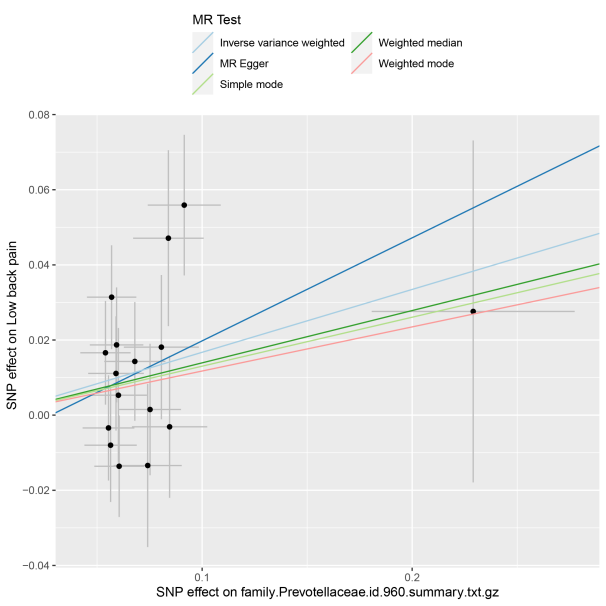
**

C
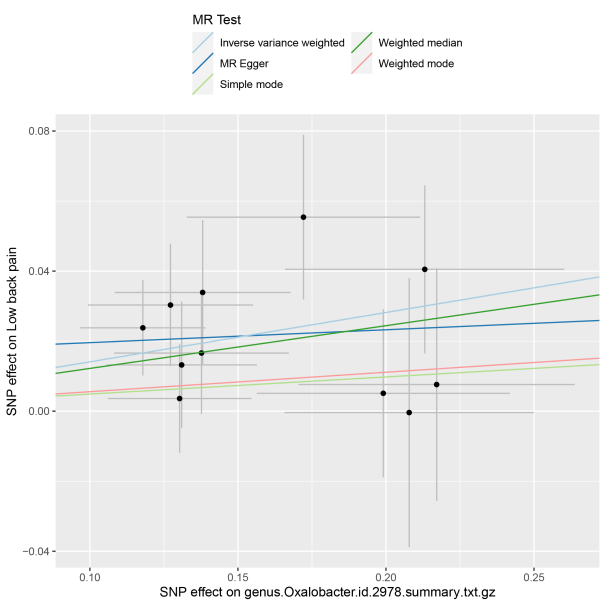
D
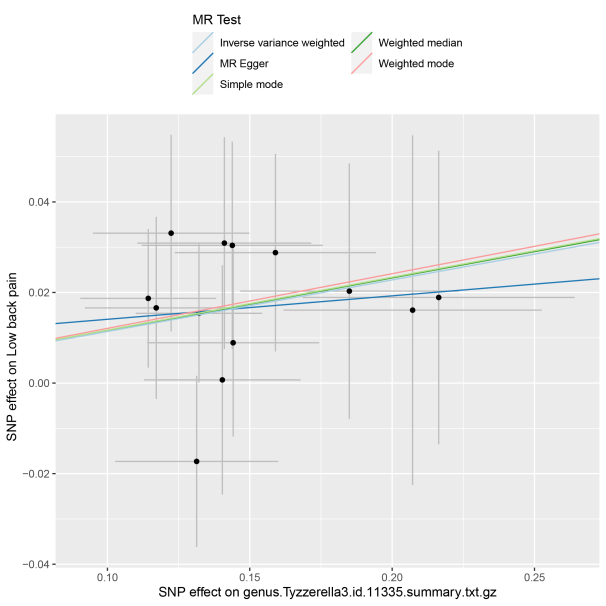


**Supplementary Figure S7.**

Scatter plots of causal estimates of exposure (Specific gut microbiota) on rheumatoid arthritis. The slope of each line corresponding to the estimated MR effect in different models, including the conventional IVW, Weighted median, MR-Egger, Simple mode, and Weighted mode. (A): *Class Clostridia*; (B): *Family Christensenellaceae* ;(C): *Family* *ClostridialesvadinBB60group*; (D): *Family Desulfovibrionaceae*; (E): *Family.Oxalobacteraceae*; (F): *Family Streptococcaceae*; (G): *Genus Desulfovibrio*; (H): *Genus Oxalobacter*; (I): G*enus RuminococcaceaeUCG002*; (J): *Genus RuminococcaceaeUCG013*;(K): *Genus Turicibacter*; (L): *Order Bacillales* ; (M)*Order Clostridiales* ; (N): *Phylum Cyanobacteria*

A
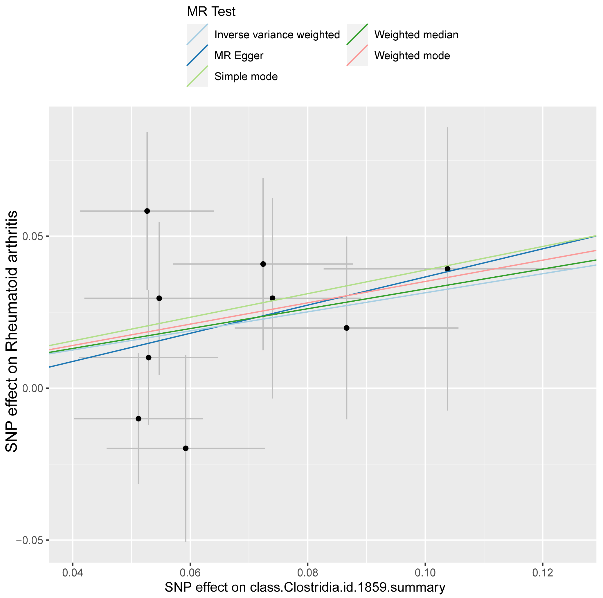
B
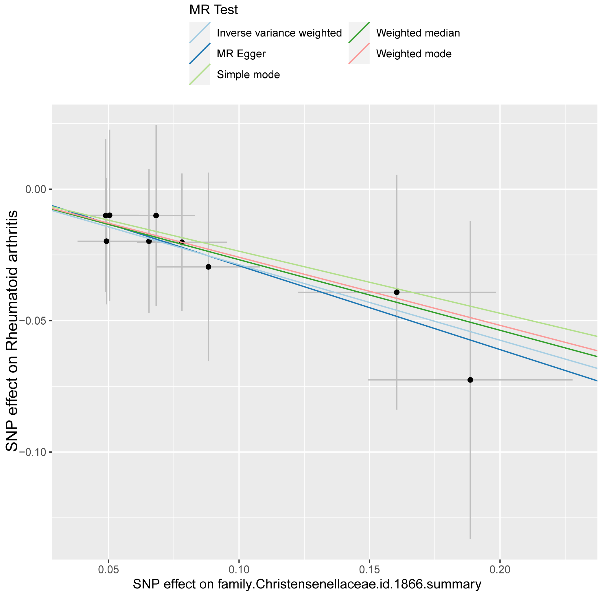


C
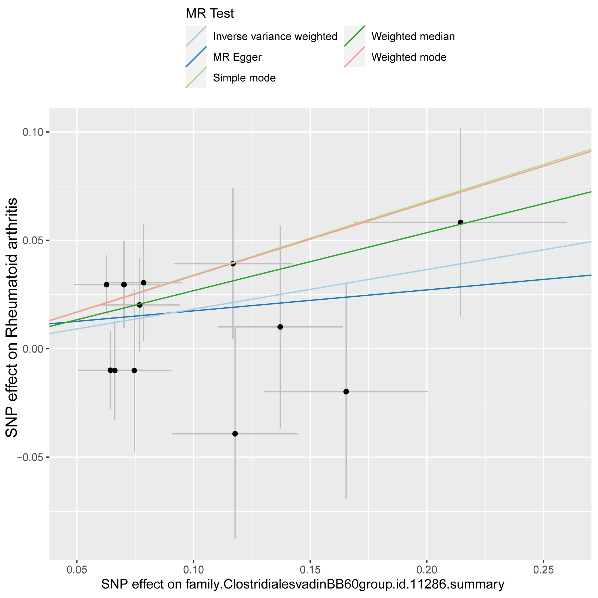
D
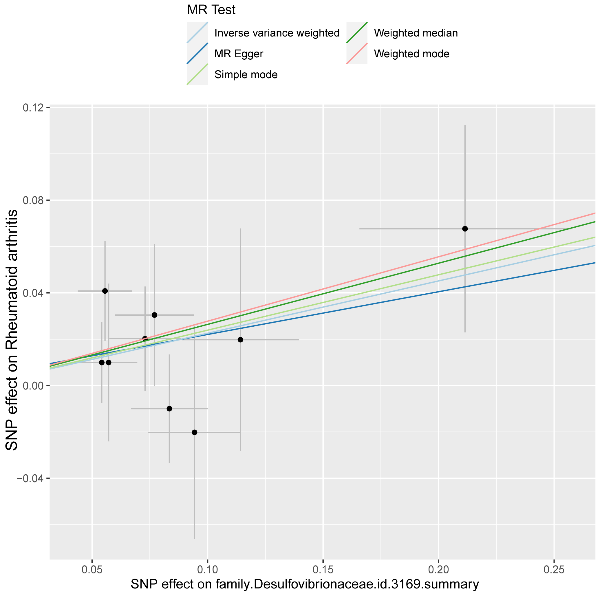


E
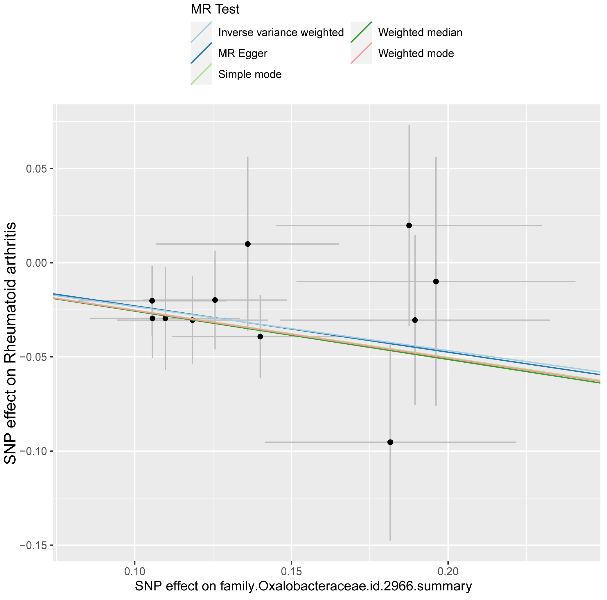
F
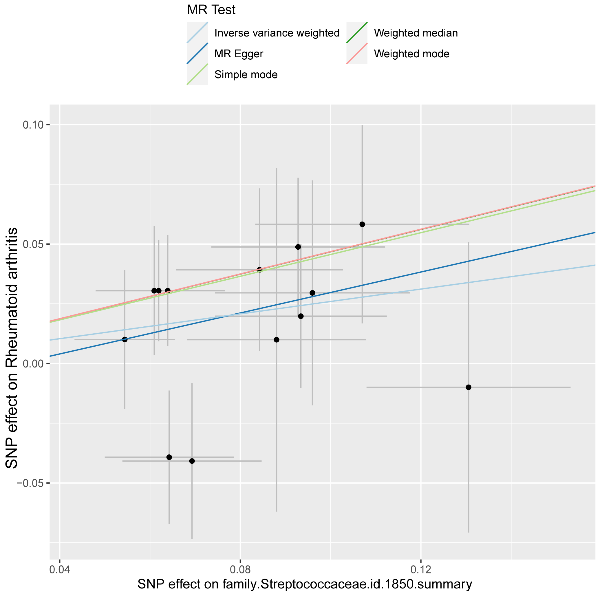


G
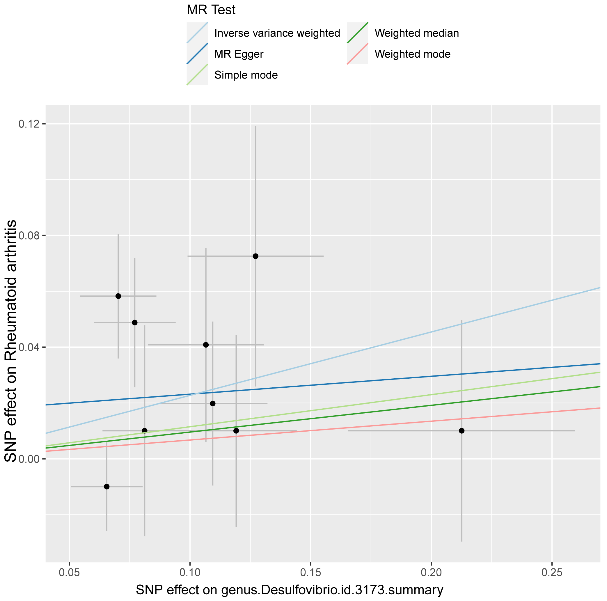
H
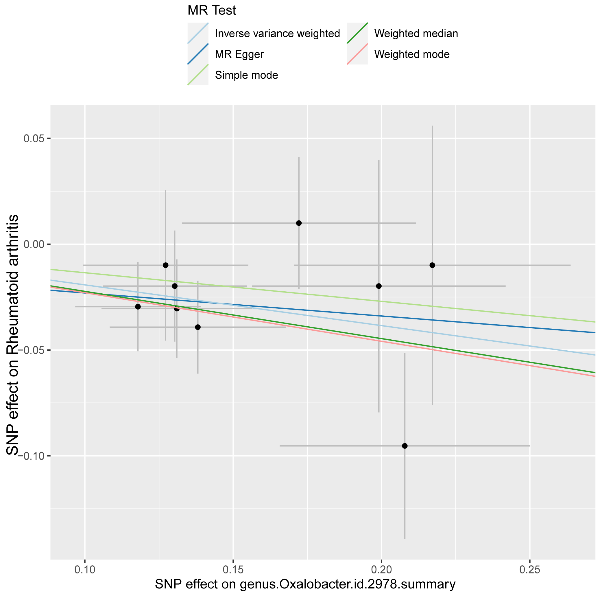


I
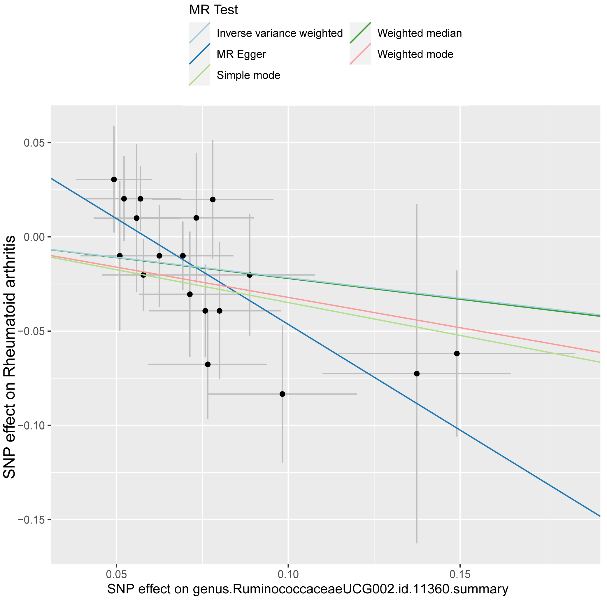
J
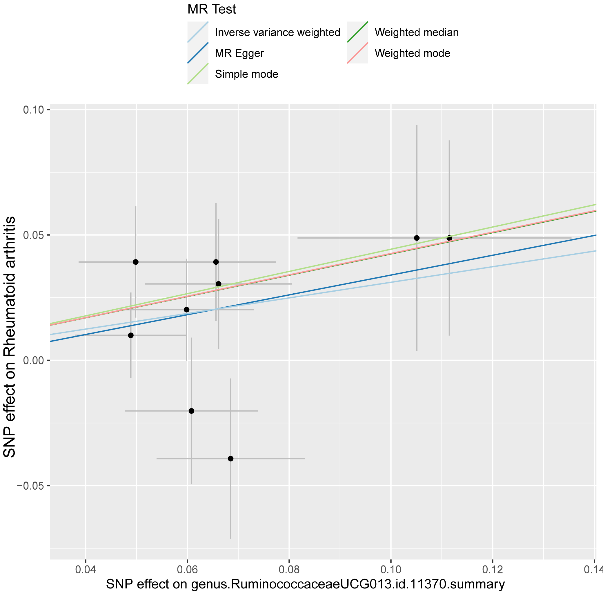


K
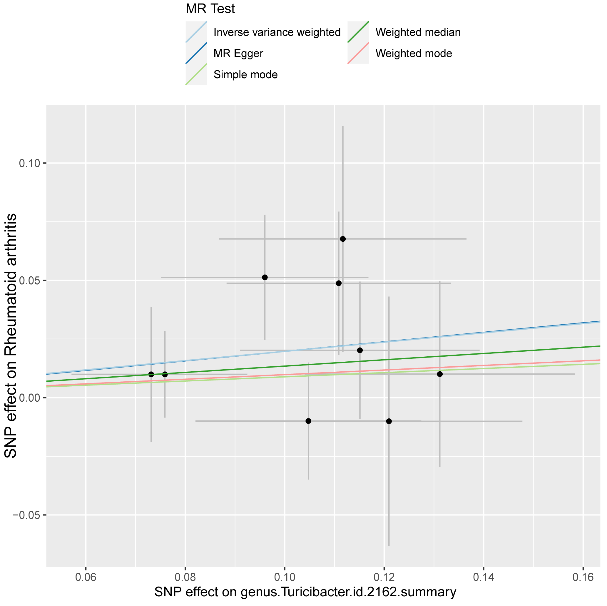
L
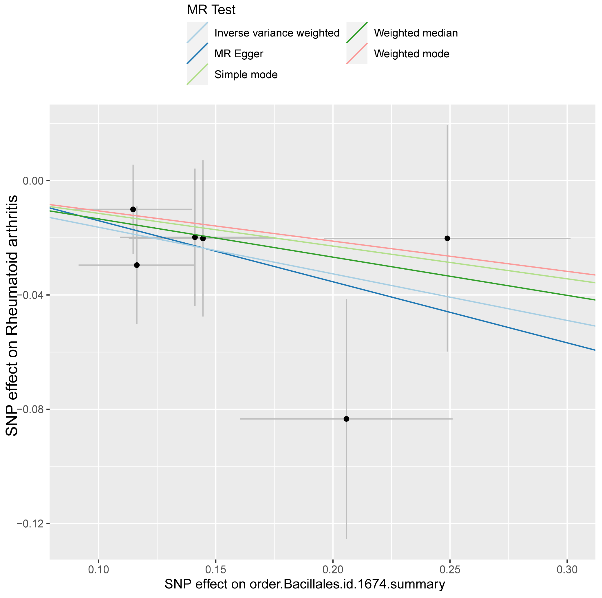


M
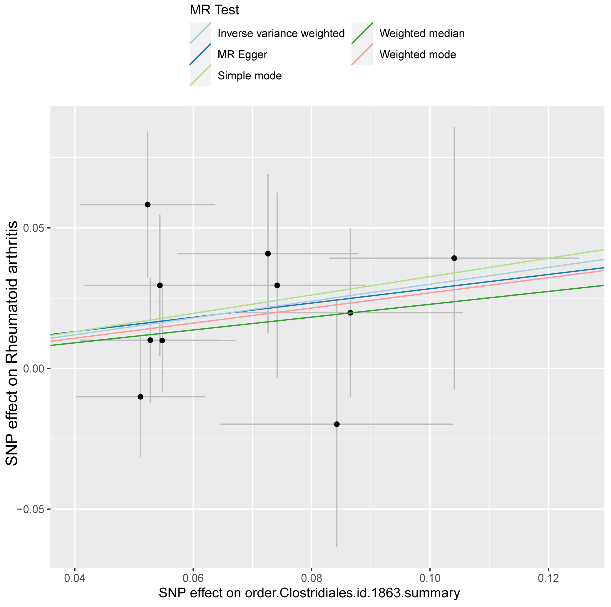
N
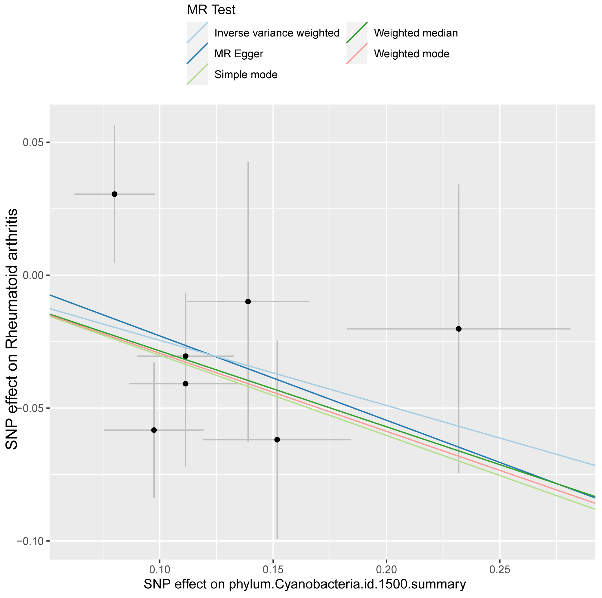


**Supplementary Figure S8.**

Scatter plots of causal estimates of exposure (Specific gut microbiota) on ankylosing spondylitis. The slope of each line corresponding to the estimated MR effect in different models, including the conventional IVW, Weighted median, MR-Egger, Simple mode, and Weighted mode. (A): *Family Lactobacillaceae*; (B): *Family Rikenellaceae*; (C): *Genus RuminococcaceaeNK4A214group*; (D): *Genus Howardella*; (E): *Genus Anaerotruncus*

A
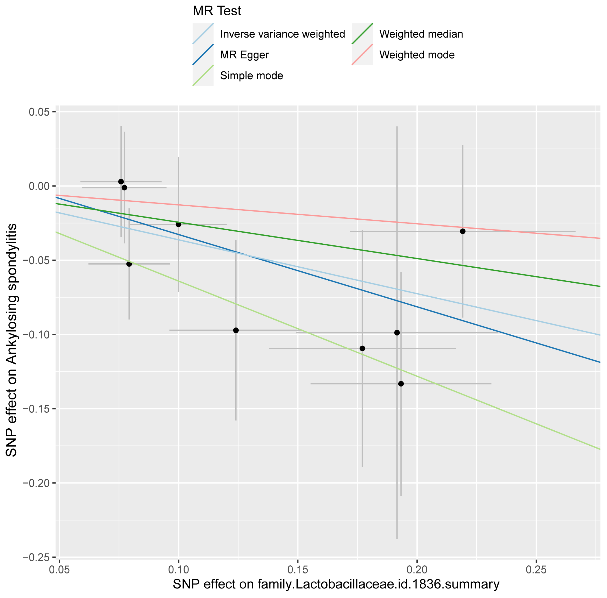
B*
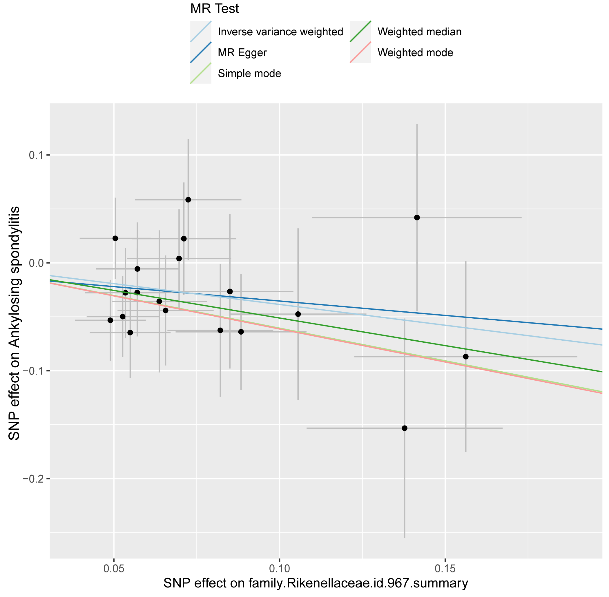
*

C
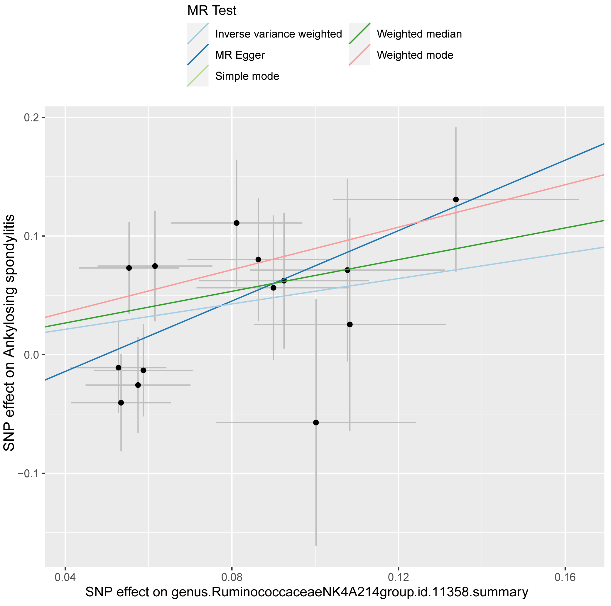
D
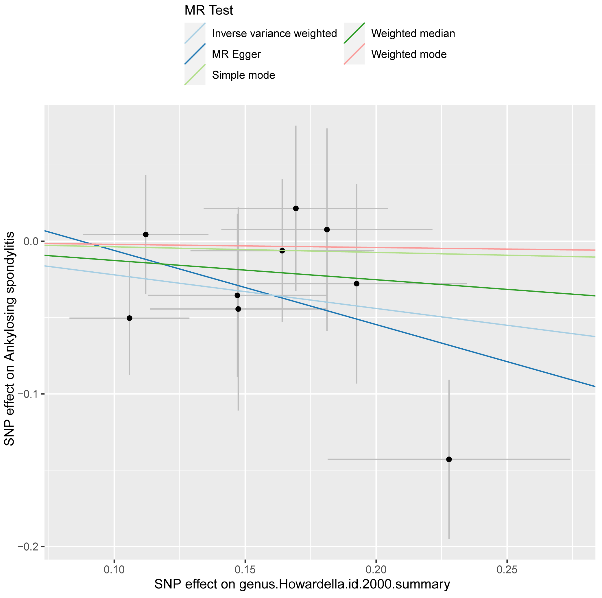


E
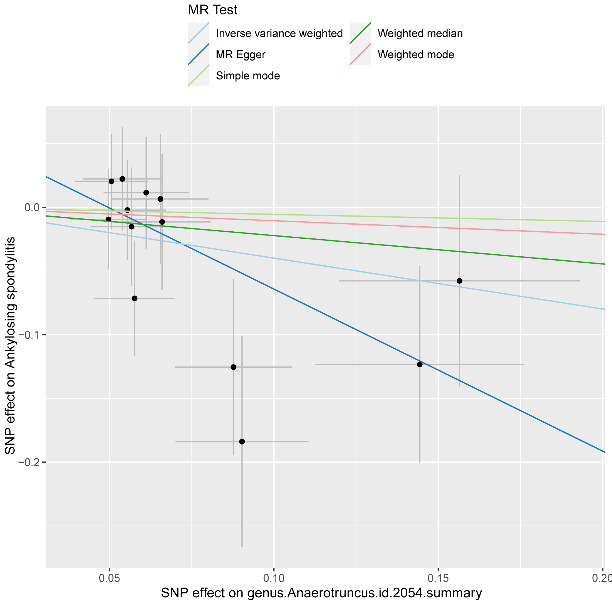


**Supplementary Figure S9.**

Leave-one-out stability tests causal estimates of exposure (Specific gut microbiota) on osteoporosis. Calculate the MR results of the remaining IVs after removing the IVs one by one.(A): *Order NB1n*; (B): *Genus LachnospiraceaeNK4A136group*; (C): *Genus Howardella*; (D): *Genus ChristensenellaceaeR.7group*; (E): *Genus Eubacteriumoxidoreducensgroup*;

A
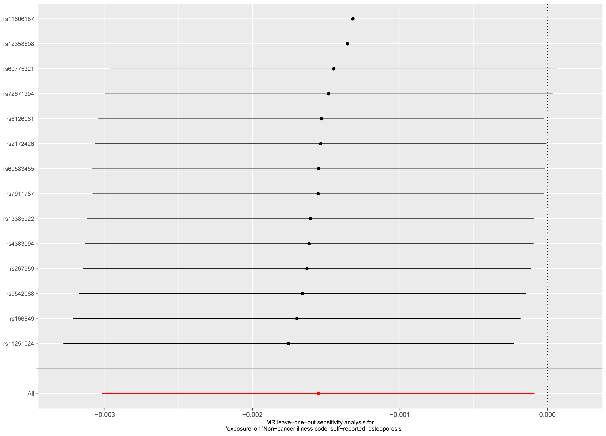
B
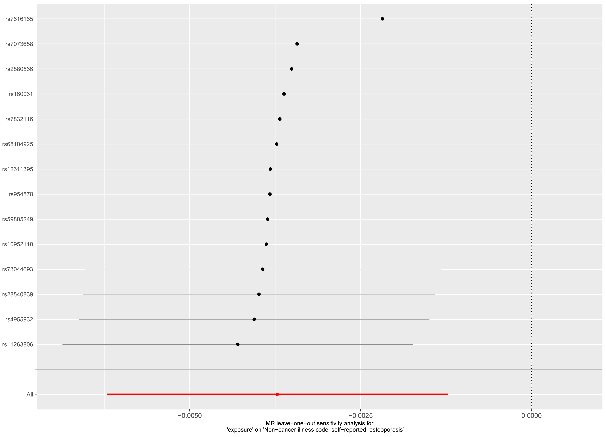


C
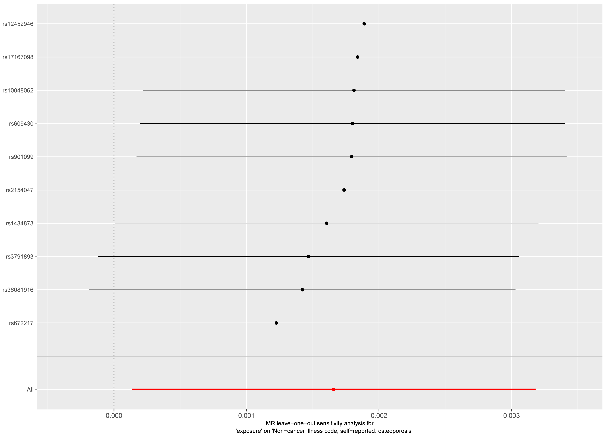
D
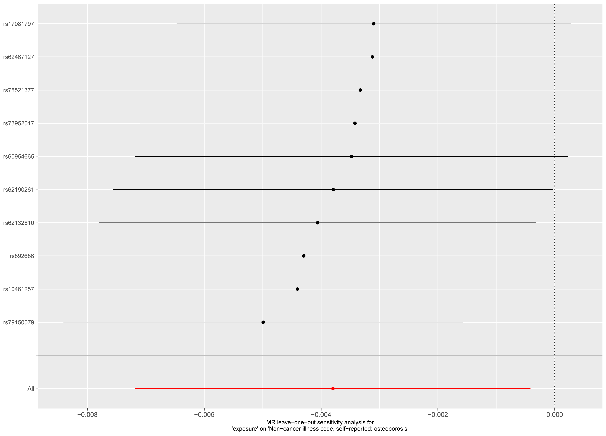


E
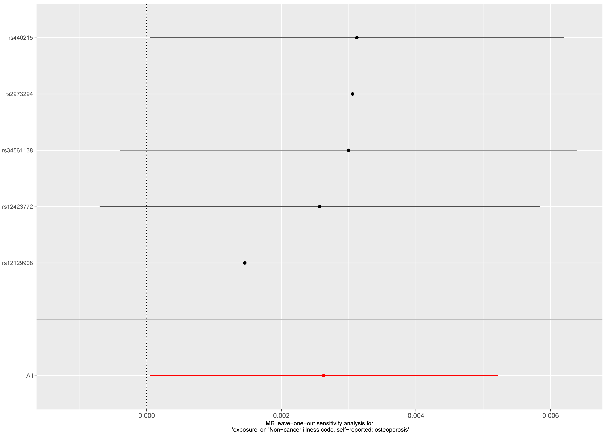


**Supplementary Figure S10.**

Leave-one-out stability tests causal estimates of exposure (Specific gut microbiota) on fracture. Calculate the MR results of the remaining IVs after removing the IVs one by one. (A): *Class Mollicutes*; (B): *Family Defluviitaleaceae*; (C): *Family BacteroidalesS24.7group*; (D): *Genus Allisonella*; (E): *Genus Collinsella*; (F): *Genus DefluviitaleaceaeUCG011*; (G): *Phylum Tenericutes*

A
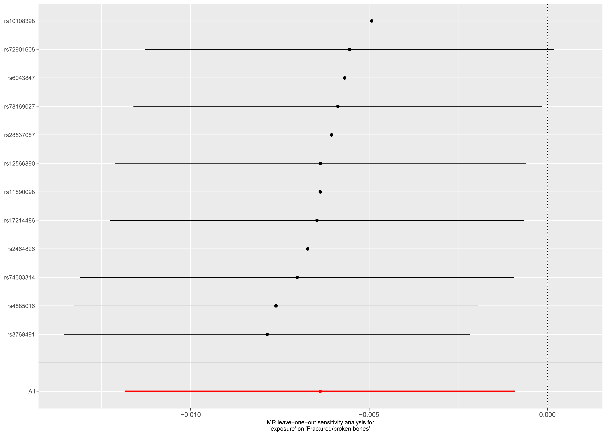
B
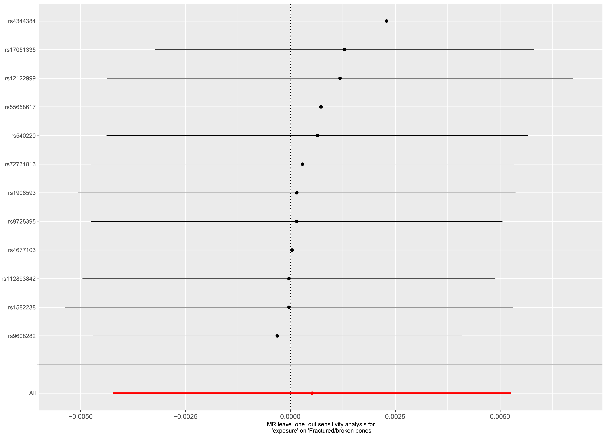


C
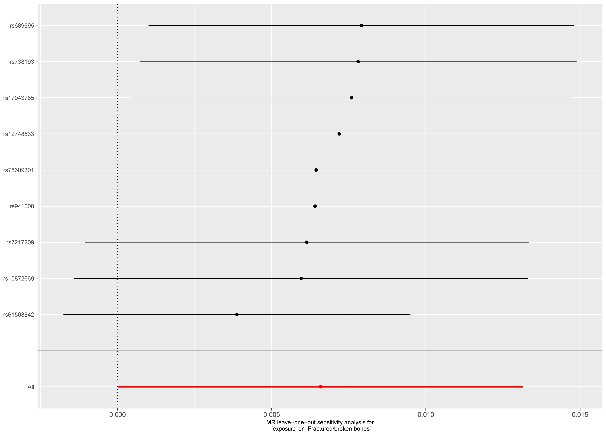
D
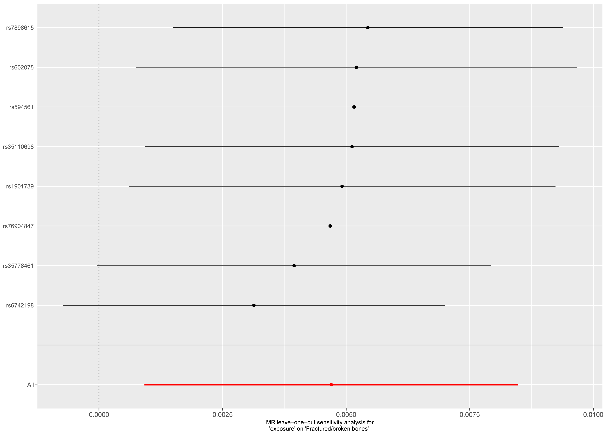


E
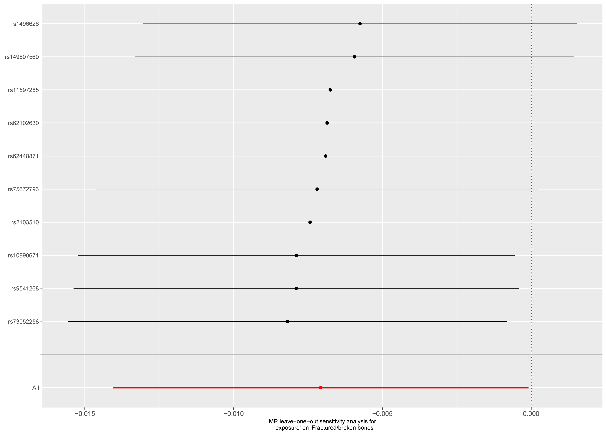
F
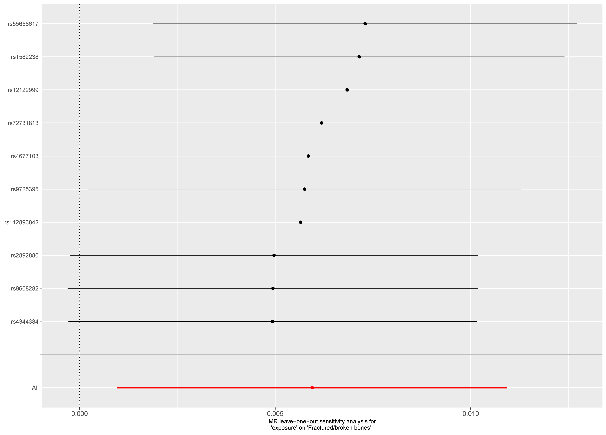


G
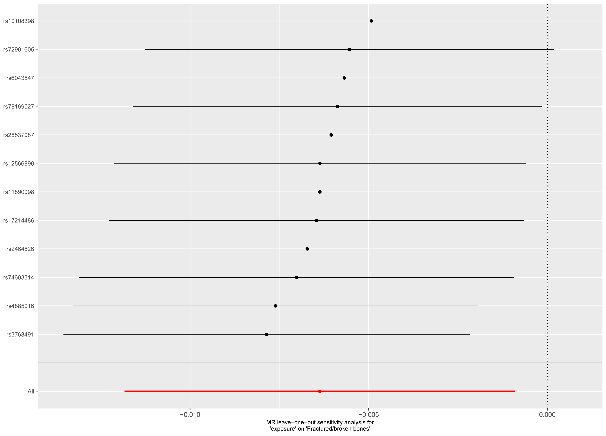


**Supplementary Figure S11.**

Leave-one-out stability tests causal estimates of exposure (Specific gut microbiota) on hand grip strength (Right). Calculate the MR results of the remaining IVs after removing the IVs one by one. (A): *Class Actinobacteria*; (B): *Family Bifidobacteriaceae*; (C): *Genus Alloprevotella*; (D): *Genus Bifidobacterium*; (E): *Genus Eisenbergiella*; (F): *Genus Parabacteroides;* (G): *Genus Paraprevotella*; (H): *Genus Prevotella9*; (I): *Genus Sellimonas*; (J): *Order Bifidobacteriales*; (K): *Phylum Actinobacteria*;

A
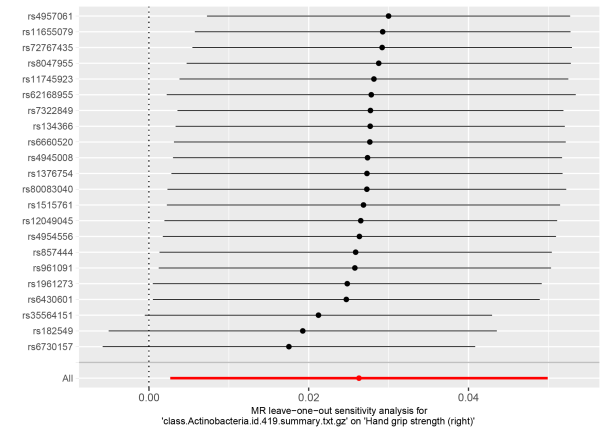
B
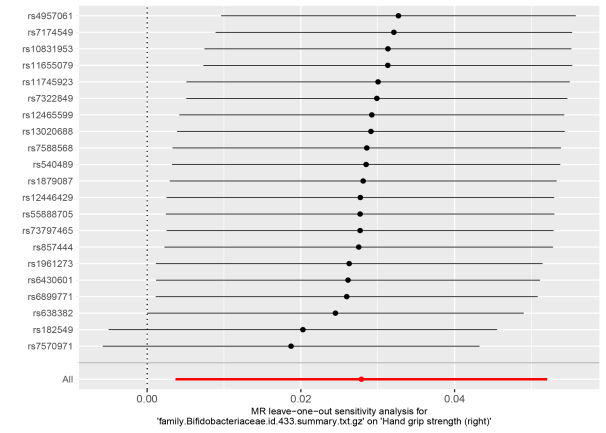


C
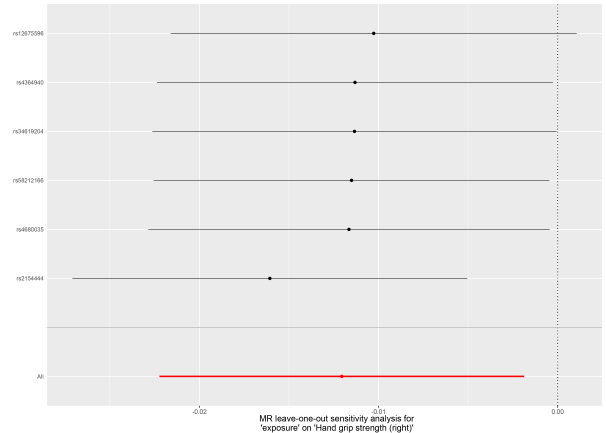
D
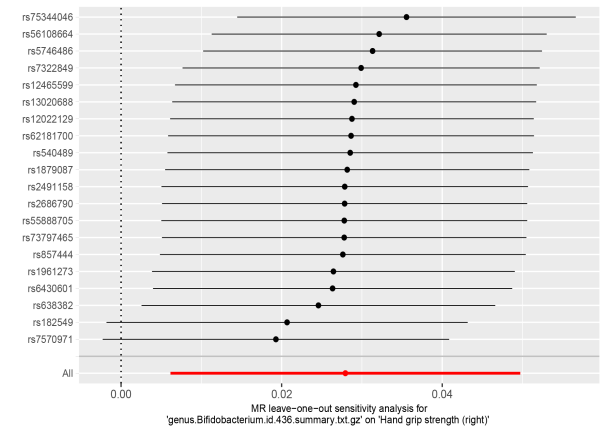


E
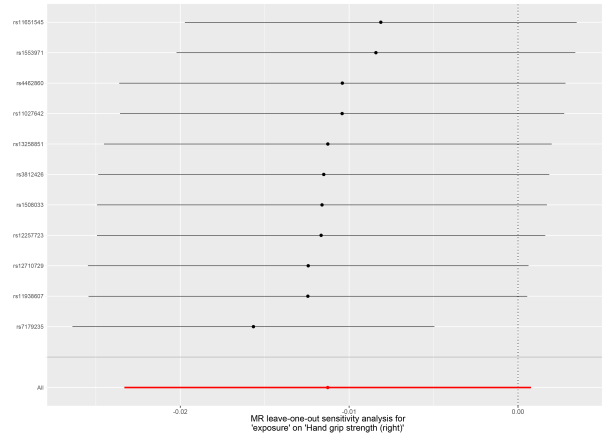
F
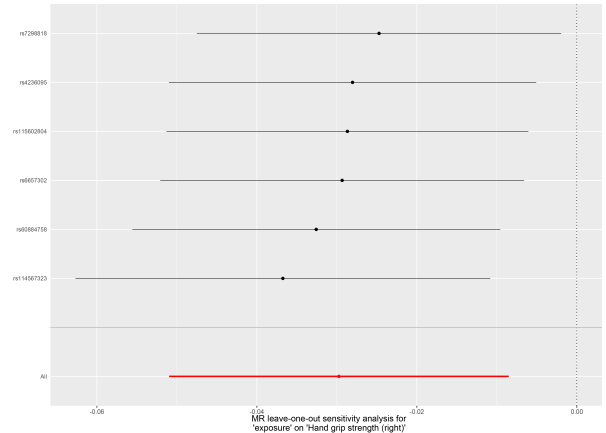


G
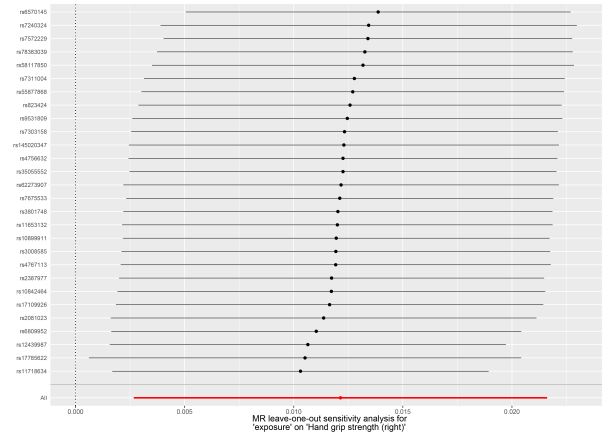
H
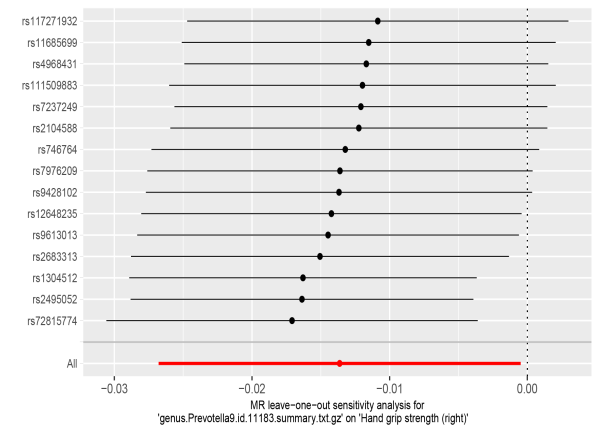
I
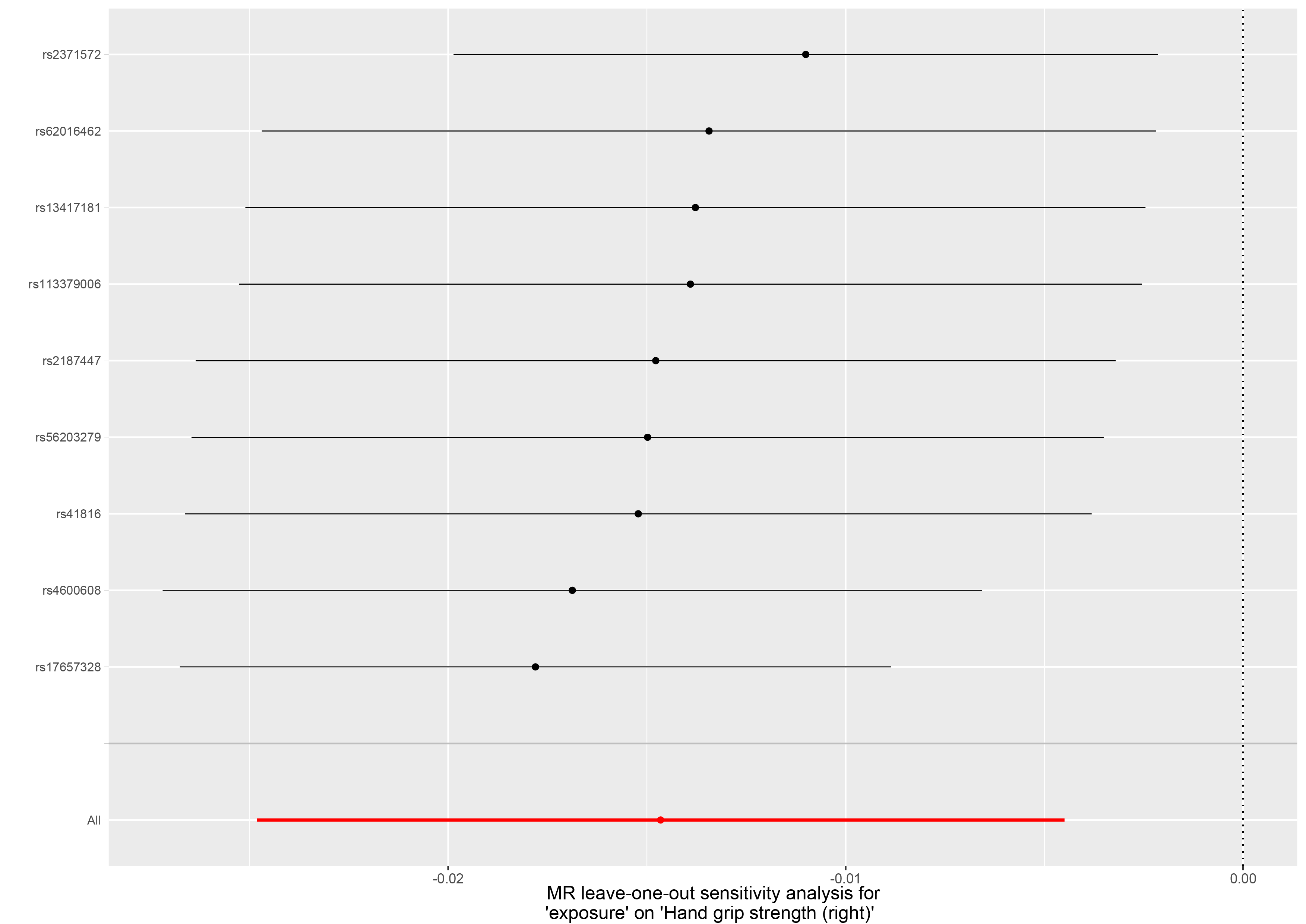
J
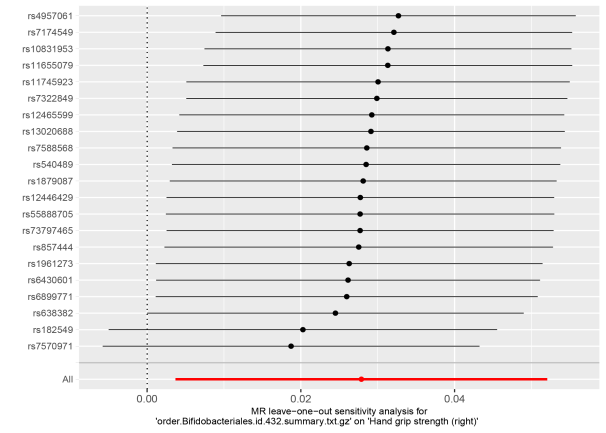


K
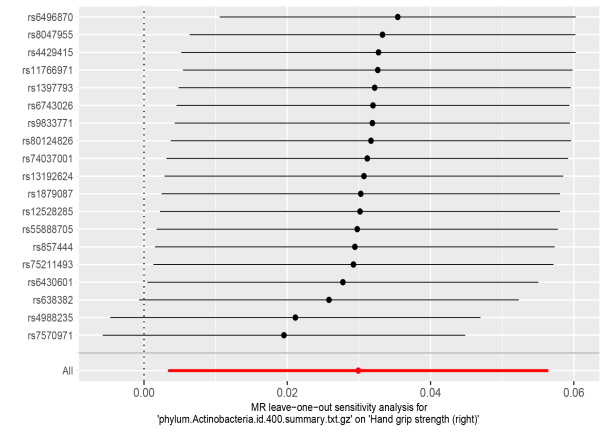


**Supplementary Figure S12.**

Leave-one-out stability tests causal estimates of exposure (Specific gut microbiota) on hand grip strength (Left). Calculate the MR results of the remaining IVs after removing the IVs one by one. (A): *Family Bifidobacteriaceae*; (B): *Genus Eubacterium nodatum group*; (C): *Genus Bifidobacterium*; (D): *Genus Olsenella*; (E): *Genus Parabacteroides*; (F): *Genus Sellimonas*; (G): *Order Bifidobacteriales*;

A**
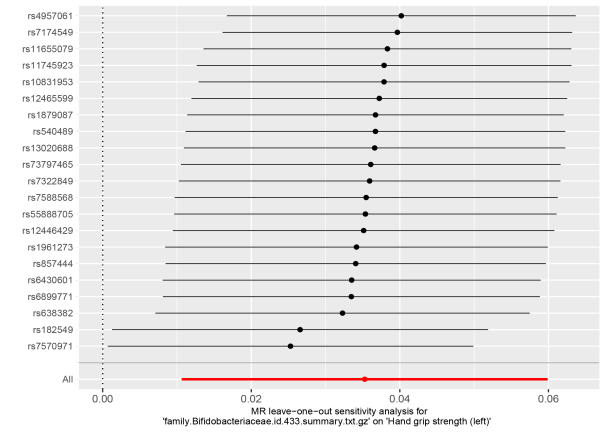
**B**
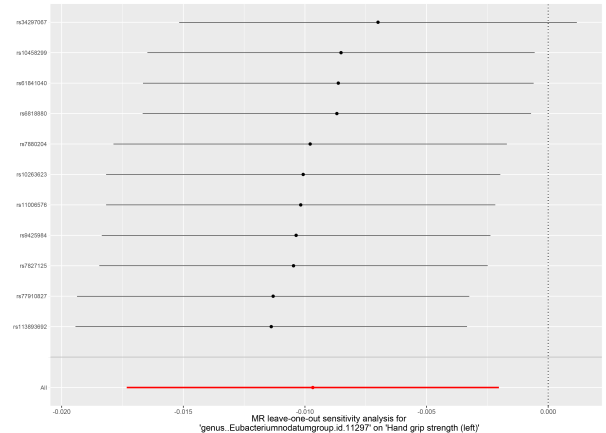
**

C
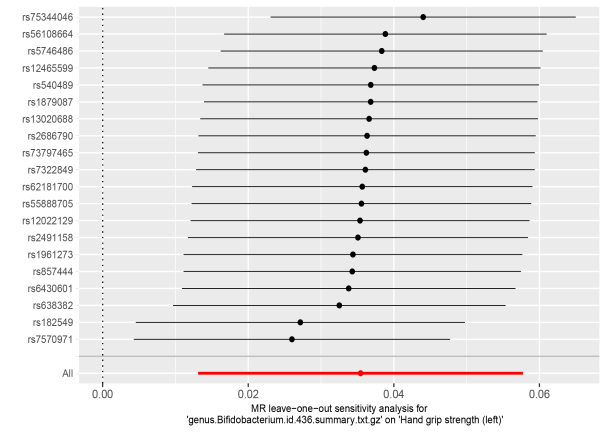
D
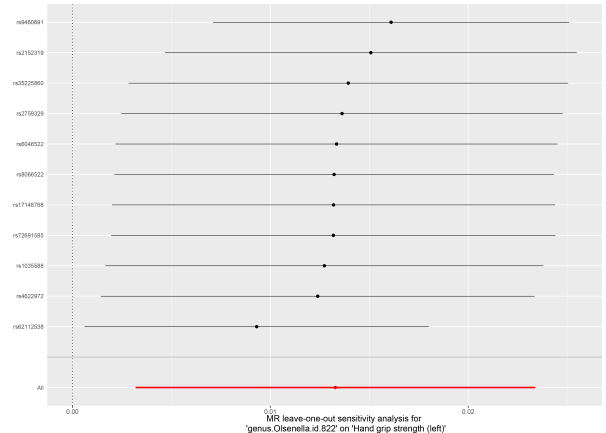


E**
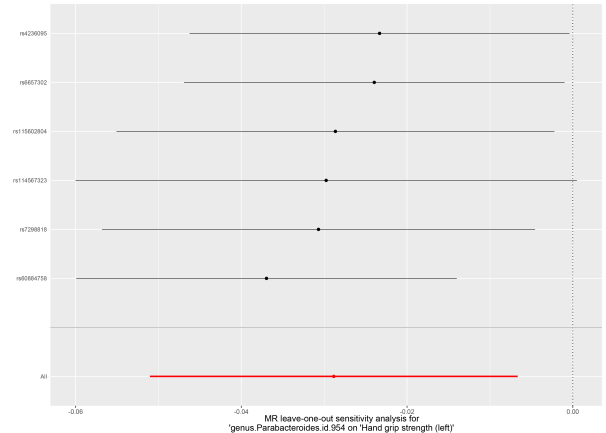
**F
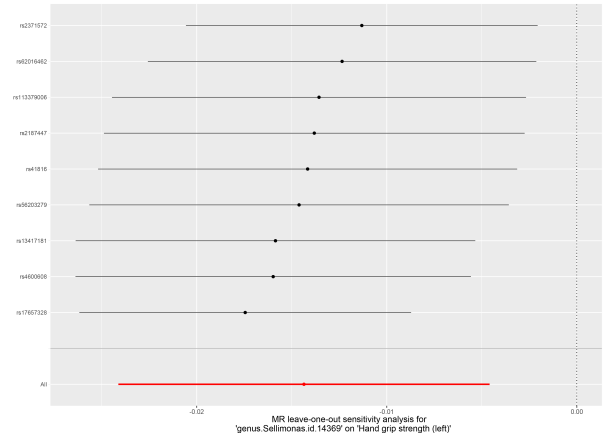


G
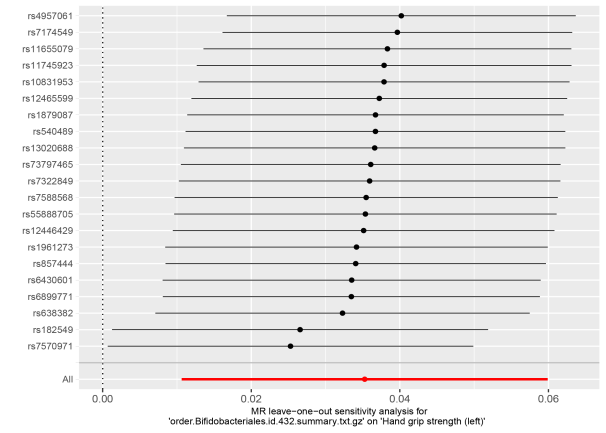


**Supplementary Figure S13.**

Leave-one-out stability tests causal estimates of exposure (Specific gut microbiota) on appendicular lean mass. Calculate the MR results of the remaining IVs after removing the IVs one by one. (A): *Family Bacteroidaceae*; (B): *Genus Eubacterium fissicatena group*; (C): *Genus Bacteroides*; (D): *Genus Lachnospira*; (E): *Genus Phascolarctobacterium*;

A**
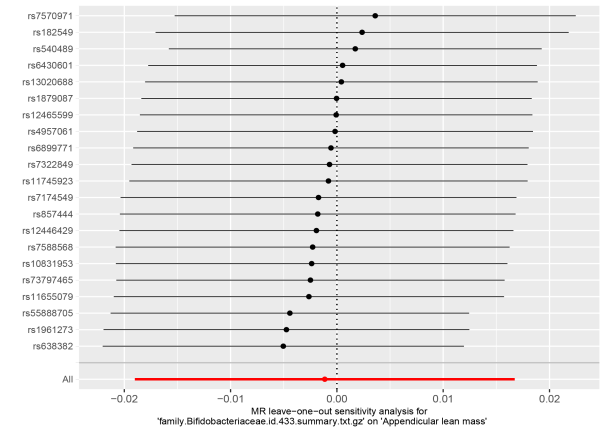
**B**
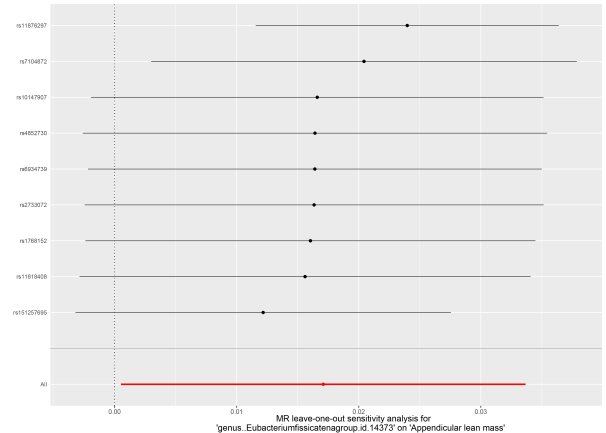
**

C
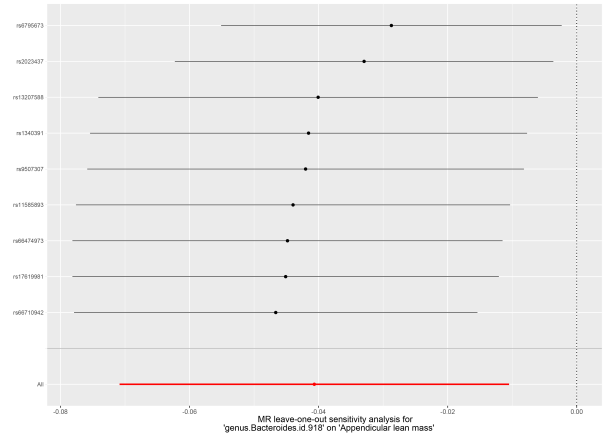
D
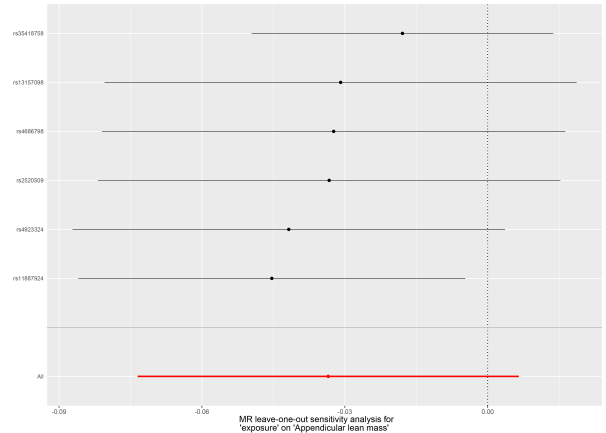


E
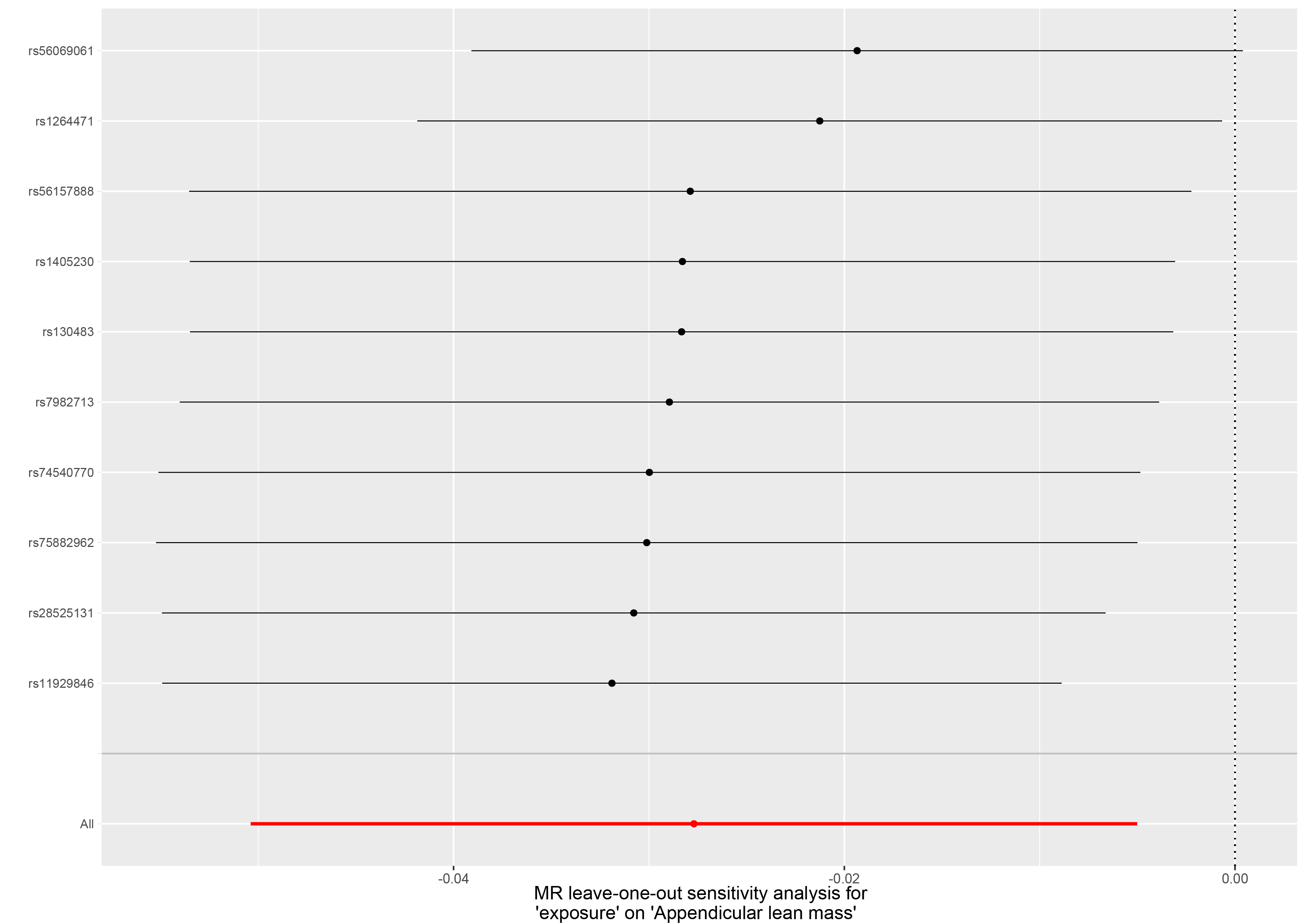


**Supplementary Figure S14.**

Leave-one-out stability tests causal estimates of exposure (Specific gut microbiota) on low back pain. Calculate the MR results of the remaining IVs after removing the IVs one by one. (A): *Class Melainabacteria*; (B): *Family Prevotellaceae*;(C): *Genus Oxalobacter*; (D): *Genus Tyzzerella3*

A**
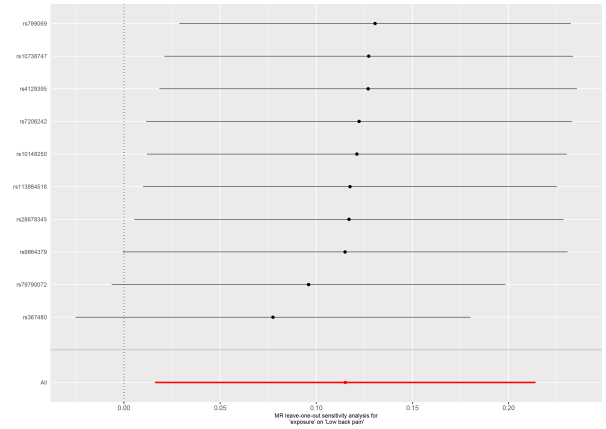
**B
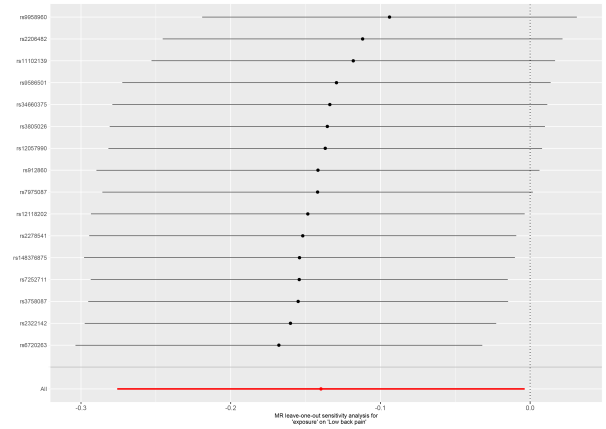


C
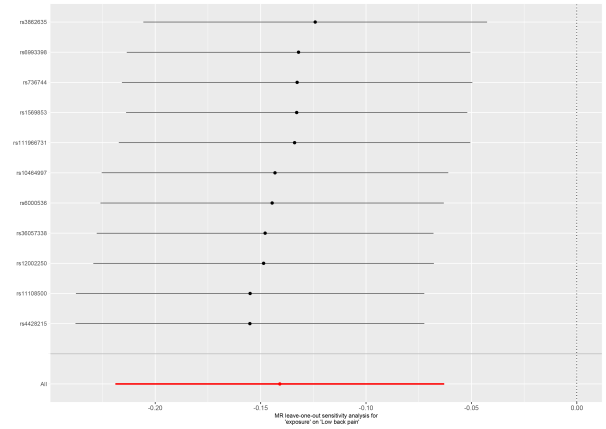
D
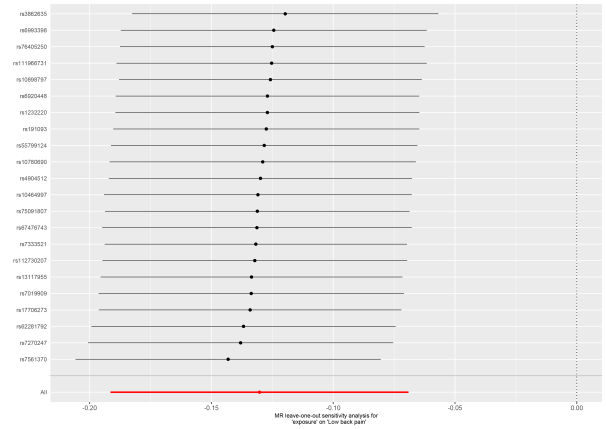


**Supplementary Figure S15.**

Leave-one-out stability tests causal estimates of exposure (Specific gut microbiota) on rheumatoid arthritis. Calculate the MR results of the remaining IVs after removing the IVs one by one. (A): *Class Clostridia*; (B): *Family Christensenellaceae* ;(C): *Family ClostridialesvadinBB60group*; (D): *Family Desulfovibrionaceae*; (E): *Family.Oxalobacteraceae*; (F): *Family Streptococcaceae*; (G): *Genus Desulfovibrio*; (H): *Genus Oxalobacter*; (I): G*enus RuminococcaceaeUCG002* ;(J): *Genus RuminococcaceaeUCG013*;(K)*Genus Turicibacter*; (L)*Order Bacillales* ; (M)*Order Clostridiales* ; (N)*Phylum Cyanobacteria*

A
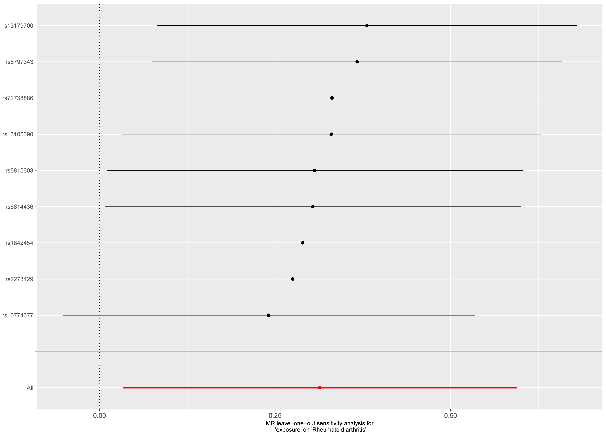
B*
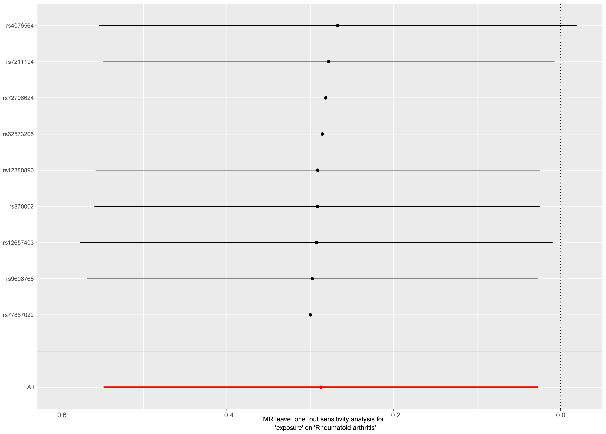
*

C
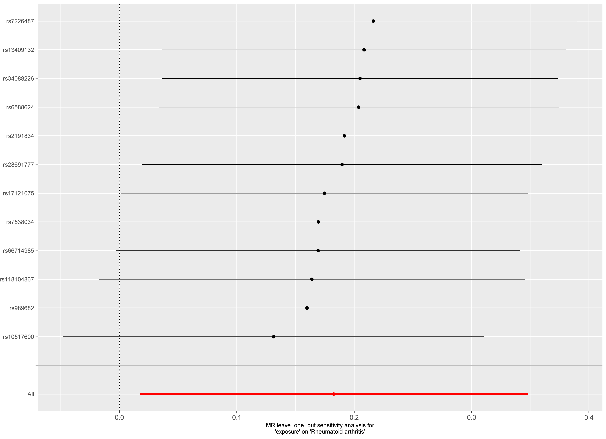
D

EF

GH

IJ

KL

MN

**Supplementary Figure S16.**

Leave-one-out stability tests causal estimates of exposure (Specific gut microbiota) on ankylosing spondylitis. Calculate the MR results of the remaining IVs after removing the IVs one by one. (A): *Family Lactobacillaceae*; (B): *Family Rikenellaceae*; (C): *Genus RuminococcaceaeNK4A214group*; (D): *Genus Howardella*; (E): *Genus Anaerotruncus*

AB

CD

E
